# Host-specific functional compartmentalization within the oligopeptide transporter during the *Borrelia burgdorferi* enzootic cycle

**DOI:** 10.1371/journal.ppat.1009180

**Published:** 2021-01-11

**Authors:** Ashley M. Groshong, Melissa A. McLain, Justin D. Radolf

**Affiliations:** 1 Department of Medicine, UConn Health, Farmington, Connecticut, United States of America; 2 Department of Pediatrics, UConn Health, Farmington, Connecticut, United States of America; 3 Department of Molecular Biology and Biophysics, UConn Health, Farmington, Connecticut, United States of America; 4 Department of Genetics and Genome Science, UConn Health, Farmington, Connecticut, United States of America; 5 Department of Immunology, UConn Health, Farmington, Connecticut, United States of America; Medical College of Wisconsin, UNITED STATES

## Abstract

*Borrelia burgdorferi* must acquire all of its amino acids (AAs) from its arthropod vector and vertebrate host. Previously, we determined that peptide uptake via the oligopeptide (Opp) ABC transporter is essential for spirochete viability *in vitro* and during infection. Our prior study also suggested that *B*. *burgdorferi* employs temporal regulation in concert with structural variation of oligopeptide-binding proteins (OppAs) to meet its AA requirements in each biological niche. Herein, we evaluated the contributions to the *B*. *burgdorferi* enzootic cycle of three of the spirochete’s five OppAs (OppA1, OppA2, and OppA5). An *oppA1* transposon (*tn*) mutant lysed in the hyperosmolar environment of the feeding tick, suggesting that OppA1 imports amino acids required for osmoprotection. The *oppA2tn* mutant displayed a profound defect in hematogenous dissemination in mice, yet persisted within skin while inducing only a minimal antibody response. These results, along with slightly decreased growth of the *oppA2tn* mutant within DMCs, suggest that OppA2 serves a minor nutritive role, while its dissemination defect points to an as yet uncharacterized signaling function. Previously, we identified a role for OppA5 in spirochete persistence within the mammalian host. We now show that the *oppA5tn* mutant displayed no defect during the tick phase of the cycle and could be tick-transmitted to naïve mice. Instead of working in tandem, however, OppA2 and OppA5 appear to function in a hierarchical manner; the ability of OppA5 to promote persistence relies upon the ability of OppA2 to facilitate dissemination. Structural homology models demonstrated variations within the binding pockets of OppA1, 2, and 5 indicative of different peptide repertoires. Rather than being redundant, *B*. *burgdorferi*’s multiplicity of Opp binding proteins enables host-specific functional compartmentalization during the spirochete lifecycle.

## Author summary

We have previously demonstrated that *Borrelia burgdorferi*, the causative agent of Lyme disease, is uniquely dependent on peptide uptake within its arthropod vector and mammalian host. The multi-component oligopeptide (Opp) uptake system responsible for peptide acquisition is intricately regulated to meet the bacterium’s nutritional needs within its ever-changing environmental niches. Herein, we demonstrate that three of the five encoded oligopeptide binding proteins (OppA1, OppA2, and OppA5), the subunits that deliver peptide ligands to the cytoplasmic membrane permease, function within different stages of the enzootic cycle (tick versus mammalian host). Lack of OppA1 results in lysis of spirochetes during tick feeding; we propose that OppA1 plays a role in osmoprotection by importing AAs protective against cell lysis in the varying osmolality of the tick midgut environment. The *oppA2* mutant has a profound dissemination defect in mice, a process that is essential for maintenance of the enzootic cycle. The *oppA5* mutant, which we previously demonstrated promotes persistence within the mammalian host, is dispensible for the tick phases of the enzootic cycle.

## Introduction

Bacteria utilize varying combinations of synthesis and acquisition to meet their nutritional requirements, contingent on their biosynthetic capabilities and the availability of nutrients in a given environment [1]. During infection, pathogenic bacteria must compete with the host for sequestered and/or scarce nutrients (*e*.*g*., transition metals), a concept now recognized as ‘nutritional virulence’ [1]. Bacteria transmitted by arthropods often have highly reduced genomes, resulting in loss of biosynthetic genes [2,3]. *Borrelia burgdorferi*, the Lyme disease (LD) spirochete, is maintained within an enzootic cycle requiring transmission between and adaptation to an arthropod vector and vertebrate reservoir host [4,5]. *B*. *burgdorferi* is an extreme auxotroph [6,7], requiring uptake of numerous components for survival, such as purines [8], cholesterol and long-chain fatty acids [9,10], and carbon sources [11–13]. In the context of LD, the concept of nutritional virulence encompasses acquisition by *B*. *burgdorferi* of a diverse array of nutrients in two vastly different, hostile milieus.

Unlike many bacteria, *B*. *burgdorferi* encodes no pathways for *de novo* synthesis of amino acids (AAs) [7,14]. Instead, the spirochete utilizes a handful of annotated free AA transporters, along with an elaborate oligopeptide (Opp) ABC transporter, to procure AAs from the reservoir host and arthropod vector [6,7,14–20]. The Opp system consists of five substrate-binding proteins (SBPs), OppA1-3 (BB0328-330), OppA4 (BBB16), and OppA5 (BBA34); a presumptive ‘primary’ permease, OppB1C1 (BB0332-333), and a heterodimeric ATPase, OppDF (BB0334-335) [7,14,19,20]. In addition, the *B*. *burgdorferi* genome encodes an ‘orphan’ permease (BB0746-747), annotated in UniProt as ‘oligopeptide transport system permease protein OppB/C.’ By generating a conditional mutant containing an inducible *oppDF* (ATPase^cond^), we recently demonstrated that peptide uptake via the Opp system is essential for viability of *B*. *burgdorferi in vitro* and murine infectivity [14]. Thus, *B*. *burgdorferi* differs from other bacterial species that employ Opp systems as an ancillary means of acquiring AAs [21].

Though ABC transporter SBPs typically engage their ligands via side chain-specific interactions within the binding pocket, bacterial OppAs bind in a sequence-independent manner via the peptide backbone [22–25]. *B*. *burgdorferi* OppAs use this same binding mechanism with variations in cavity volume, configuration, and electrostatics [14,26], theoretically enabling each OppA to accommodate a unique repertoire of peptides. Our previous study suggests that *B*. *burgdorferi* couples structural diversity with differential expression of OppAs to optimize peptide uptake at each phase of its enzootic cycle [14,17,27–33]. *oppA1*, *oppA2*, *oppA3* and *oppA4* are expressed within the feeding tick, while *oppA2* and *oppA5* are predominantly expressed in the mammal [14]. Evidence to date suggests that this intricate regulatory scheme involves all three of the LD spirochete’s known global regulatory networks [5,12,34–36]. Rel_Bb_ generates the alarmone, (p)ppGpp, which regulates the stringent response within the tick and promotes expression of *oppA1* and *oppA3* [27,30]. The alternative sigma factor RpoS promotes global transcriptional changes needed for mammalian host-adaptation, including positive regulation of *oppA5* [29,32,33]. Activation of the Hk1/Rrp1 two-component system during the blood meal results in the production of c-di-GMP, inducing the expression of tick phase genes, including *oppA4* [37]. Conversely, c-di-GMP represses *oppA5*, potentially explaining why this RpoS-dependent gene is expressed only during the mammalian phase [14,37].

Herein, we further explored how individual oligopeptide-binding proteins of the *B*. *burgdorferi* Opp system maintain spirochete viability in ticks and mice. We identified sharply divergent phenotypes for *oppA1* and *oppA2* transposon (*tn*) mutants. The *oppA1tn* mutant was lysed during the tick blood meal, a phenotype that we attribute to deficient uptake of amino acids required for osmoprotection. The *oppA2tn* mutant, on the other hand, displayed a profound defect in hematogenous dissemination in mice, yet persisted within skin while inducing a minimal antibody response. We hypothesize that this phenotype represents a link between peptide uptake and chemotaxis during the mammalian phase of the cycle. Our previous study [38] identified a role for OppA5 in spirochete persistence within the mammalian host; in this report we show that the *oppA5*tn mutant displayed no defect during the tick phase of the cycle and could be transmitted. Rather than being redundant, *B*. *burgdorferi*’s multiplicity of Opp binding proteins enables host-specific functional compartmentalization during the spirochete lifecycle.

## Results

### Genetic and *in vitro* characterization of *oppA1* and *oppA2 tn* mutants

**Fig 1:**
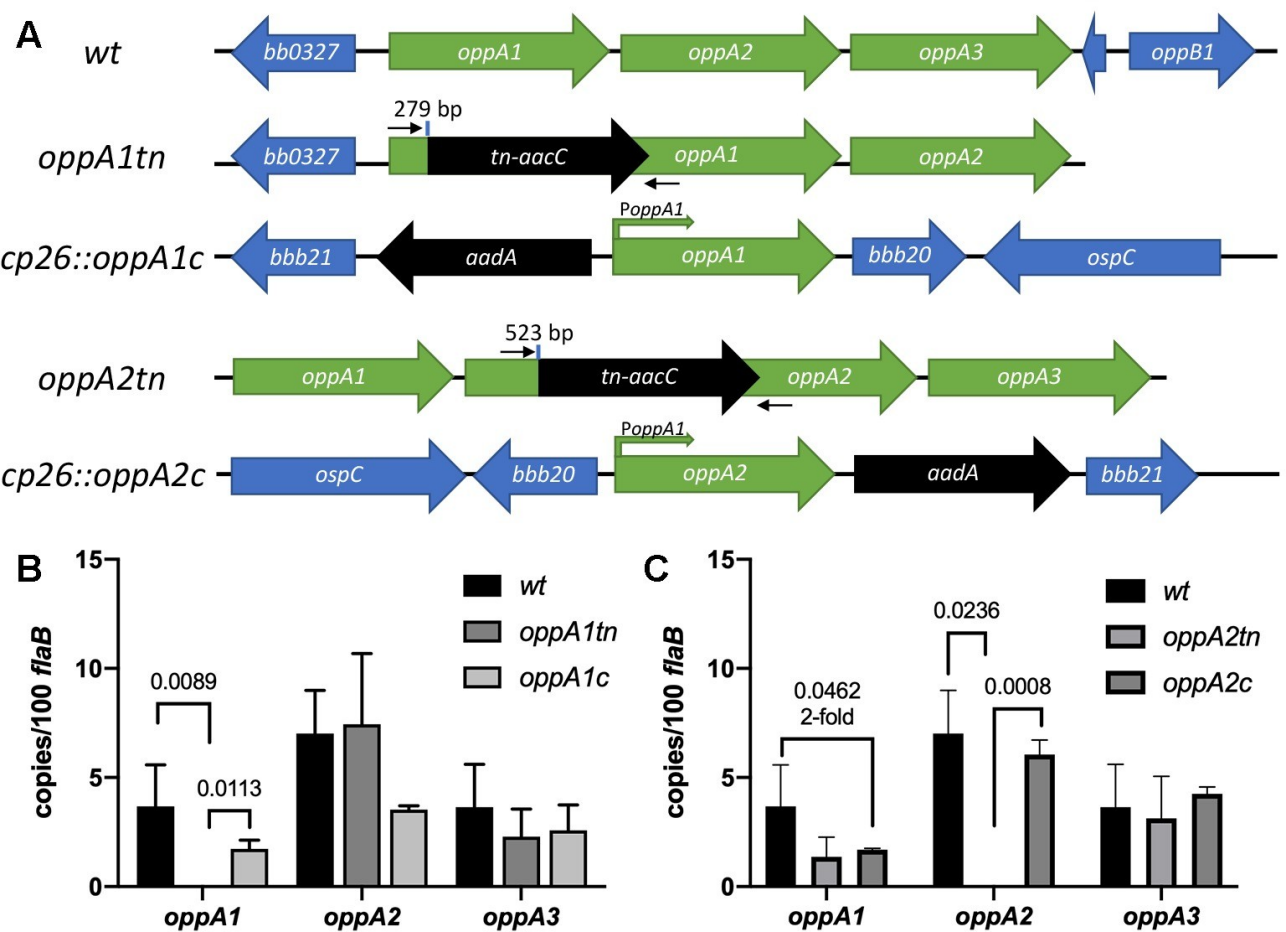
*In vitro* characterization of *oppA1* and *oppA2* mutants and complements. (A) Schematics of the *wt*, *oppA1tn* and *oppA2tn* mutants and the corresponding complements in cp26. The transposon (*tn*) insertion sites (bp of coding sequence) are indicated. *aacC* and *aadA* confer gentamycin and streptomycin resistance, respectively. qRT-PCR primers specific to the *tn* insertion site are noted with small black arrows. (B-C) Transcript copy numbers of *oppA1-3* (mean ± SEM, normalized to *flaB*) in (B) *wt*, *oppA1tn*, and *oppA1c* and (C) *wt*, *oppA2tn*, and *oppA2c* determined from triplicate biological replicates and quadruplicate technical replicates. Statistical analyses were performed using unpaired Student’s *t* test.

As noted in the Introduction, our previous expression and structural data inferred that *oppA1* and *oppA2* play distinct roles within the spirochete lifecycle [14]. Along these lines, Troy *et al*. reported that a *tn* mutant for *oppA1* was infectious in mice [39], while *oppA2tn* was attenuated [40]. We conducted experiments to further these phenotypic characterizations. We selected from the STM *tn* library [41] the *oppA1* and *oppA2 tn* mutants with the most 5’ *tn* insertions (T11TC050 and T06TC269, respectively; Fig 1A). T06TC269 was missing lp21; while the importance of this plasmid is not known, we elected to reconstruct the mutant in the *wt* strain (see Methods). We next confirmed by qRT-PCR that the *tn* insertions in *oppA1* and *oppA2* affected transcription of just the targeted genes (Fig 1B and 1C). Compared to *wt*, both mutants were morphologically indistinguishable (S1A Fig), exhibited normal motility in BSK-II (S1–S3 Movies), and demonstrated no *in vitro* growth defects (S1B Fig). Zhou *et al*. [42] recently noted that expression of OspC is dysregulated in an *oppA4* mutant; therefore, we confirmed that our mutants temperature-shifts normally (S1C Fig). For complementation, we inserted the *oppA1* or *oppA2* coding regions into cp26, each preceded by the 500 bp upstream of *oppA1*, which contains the native operonic promoter (Fig 1A) [17]. Complementation of either mutant restored transcription of their respective genes (Fig 1B and 1C).

### *oppA1* is essential for survival of *B*. *burgdorferi* in feeding ticks

**Fig 2:**
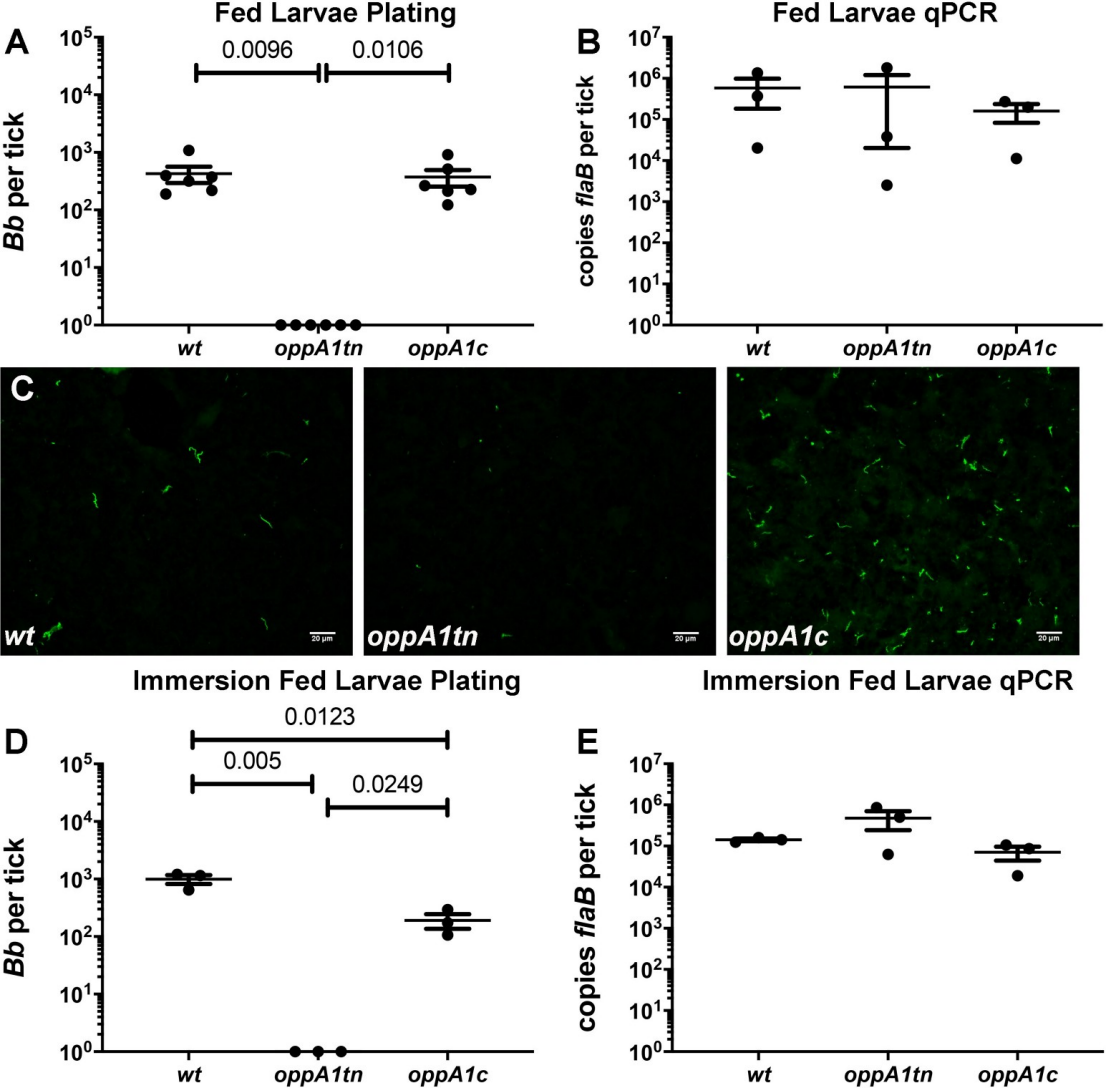
*oppA1* is essential in ticks. Spirochete burdens as assessed by (A) colony counts (mean ± SEM) and (B) qPCR in midguts of larvae naturally fed on mice infected with *wt*, *oppA1tn*, and *oppA1c*. Each data point represents a separate pool of larvae. (C) Detection of spirochetes by immunofluorescence analysis of midguts from replete larvae fed on mice infected with *wt*, *oppA1tn*, or *oppA1c* (400x total magnification). Spirochete burdens (mean ± SEM) assessed by (D) colony counts and (E) qPCR in midguts of larvae immersed in *wt*, *oppA1tn*, or *oppA1c* cultures and subsequently fed on naïve C3H/HeJ. Each data point represents a separate pool of larvae. Statistical analysis of tick studies was determined by unpaired Student’s *t* test.

To evaluate infectivity of the *oppA1tn* mutant, cohorts of mice were needle-inoculated with 1 x 10^4^ *wt* or *oppA1tn* and assessed for infection four weeks later. As previously reported [39], *oppA1tn* did not display a virulence defect (S2A Fig and Table 1 - Pilot Experiment). Two weeks after syringe-inoculation, spirochete burdens in ticks were assessed by semi-solid plating of replete larvae fed on the infected mice. In stark contrast to larvae fed on *wt*-infected mice, larvae fed on *oppA1tn*-infected mice contained no live spirochetes (S2B Fig). Mutant spirochetes also were not recovered by plating from flat nymphs after the larvae had molted (S2C Fig). These experiments were repeated with the *oppA1c* strain and, as expected, the mutant and complement were comparably infectious in mice (Table 1 –Complementation Experiment). Once again, there was no recovery of viable *oppA1tn* spirochetes from replete larvae, while complementation restored their ability to survive the larval blood meal (Fig 2A). Midguts of acquiring larvae contained equivalent bacterial burdens by qPCR (Fig 2B), confirming that *oppA1tn* was taken up at levels comparable to *wt* and complement. Immunofluorescence analysis (IFA) of midguts from larvae fed on *oppA1tn*-infected mice revealed mainly spirochete remnants (Fig 2C), which, in combination with the qPCR results, indicate that the mutant lysed in the midgut after acquisition. We used larval immersion feeding [43] to confirm that *oppA1* is essential for structural integrity of the spirochete during the blood meal. As with natural acquisition, viable mutant spirochetes were not recovered, though bacterial burdens of all three strain were indistinguishable by qPCR (Fig 2D and 2E).

**Table 1:** *oppA1* positive tissue culture data.

Strain	Ear	Inoculation Site	Tibiotarsal Joint	Bladder	Heart	Total Tissues	Total Mice
**Pilot Experiment—Needle-inoculation 10**^**4**^**–4 weeks PI**							
***wt***	10/10	10/10	10/10	10/10	10/10	50/50	10/10
***oppA1tn***	10/10	10/10	10/10	10/10	10/10	50/50	10/10
**Complementation Experiment—Needle-inoculation 10**^**4**^**–4 weeks PI**							
***wt***	7/7	7/7	7/7	7/7	7/7	35/35	7/7
***oppA1tn***	8/8	8/8	8/8	8/8	8/8	40/40	8/8
***oppA1c***	8/8	8/8	8/8	8/8	8/8	40/40	8/8

### Loss of OppA2 impairs dissemination within the mammal following needle-inoculation

In preliminary studies, mice were needle-inoculated with 1 x 10^4^ *wt* or *oppA2tn* spirochetes and evaluated by culture (pinnae, inoculation site, tibiotarsal joint, bladder, and heart) and serology four weeks later. In contrast to *oppA1tn* (Table 1), *oppA2tn* spirochetes were recovered only from the inoculation site (Table 2 –Pilot Experiment 1), and *oppA2tn*-infected mice displayed minimal serological response (S3A Fig). Complementation restored infectivity and serological responses, albeit with less robust serologies than *wt* (S3B Fig and Table 2 –Pilot Experiment 2), confirming this distinctive phenotype was due to loss of *oppA2*. Spirochete burdens in the inoculation sites were equivalent for all strains as assessed by qPCR (S3C Fig); furthermore, for all three strains, cultures from inoculation sites were darkfield-positive within the same time frame (one week). The *oppA2tn* mutant did not display a growth phenotype *in vitro* (S1B Fig) but was recovered from dialysis membrane chambers (DMCs) at ~ 4-fold lower mean final density than *wt* and *oppA2c*; this difference, while modest, was statistically significant (Table 3). Even so, DMC-cultivated *oppA2tn* host-adapted normally (i.e., downregulated OspA and upregulated OspC; S3D Fig).

**Table 2:** *oppA2* positive tissue culture data.

Strain	Ear	Inoculation Site	Tibiotarsal Joint	Bladder	Heart	Total Tissues	Total Mice
**Pilot Experiment 1—Needle-inoculation 10**^**4**^**–4 weeks PI**							
***wt***	10/10	10/10	10/10	10/10	10/10	50/50	10/10
***oppA2tn***	0/10	10/10	0/10	0/10	0/10	10/50	10/10
**Pilot Experiment 2—Needle-inoculation 10**^**4**^**–4 weeks PI**							
***wt***	5/5	5/5	5/5	5/5	5/5	25/25	5/5
***oppA2tn***	0/5	5/5	0/5	0/5	0/5	5/25	5/5
***oppA2c***	5/5	5/5	5/5	5/5	5/5	25/25	5/5
**Skin Sampling Experiment—Needle-inoculation 10**^**4**^**–4 weeks PI**							
***wt***	5/5	5/5	5/5	5/5	5/5	25/25	5/5
***oppA2tn-dorsal***	0/5	5/5	0/5	0/5	0/5	5/25	5/5
***oppA2tn-ventral***	0/5	5/5	0/5	0/5	0/5	5/25	5/5
***oppA2c***	5/5	5/5	5/5	4/5	0/5	19/25	5/5
**Nymphal Transmission Experiment—Nymph-inoculated—2 weeks PI**							
***wt***	9/10	9/10	9/10	9/10	9/10	45/50	10/10
***oppA2tn***	0/9	4/9	0/9	0/9	0/9	4/45	4/9
**Nymphal Skin Sampling Experiment—Nymph-inoculated—4 weeks PI**							
***wt***	5/5	5/5	5/5	5/5	5/5	25/25	5/5
***oppA2tn***	0/5	5/5	0/5	0/5	0/5	5/25	5/5
***oppA2c***	4/5	5/5	5/5	2/5	0/5	16/25	5/5

**Table 3:** *oppA2tn* densities from DMCs (spirochetes/ml).

Strain	Rat 1	Rat 2	Rat 3	Average (SD)	P value^a^
*wt*	1.90x10^6^	3.05x10^6^	2.05x10^6^	2.33x10^6^ (± 5.10x10^5^)	0.0125
*oppA2tn*	5.00x10^5^	8.75x10^5^	7.50x10^4^	4.83x10^5^ (± 3.27x10^5^)	na
*oppA2c*	2.28x10^6^	1.10x10^6^	2.83x10^6^	2.07x10^6^ (± 7.32x10^5^)	0.0472

a *p* value for pairwise comparison against *oppA2tn* using Student’s *t* test.

**Fig 3:**
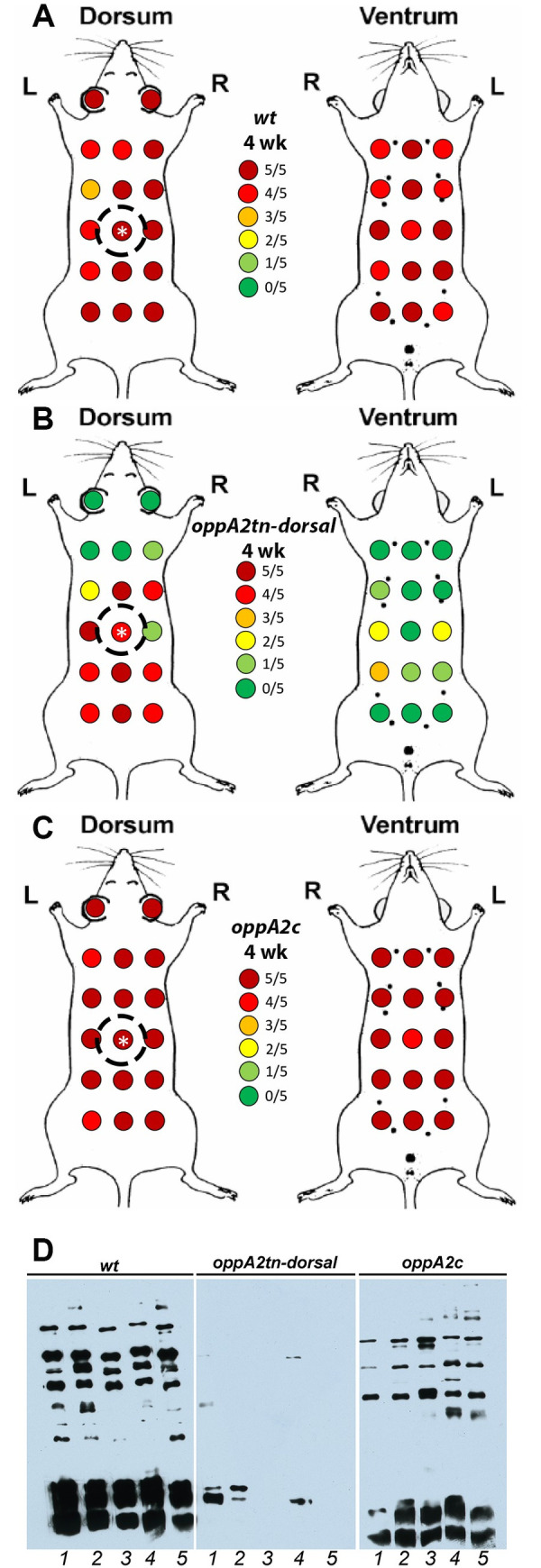
*oppA2* is essential for dissemination within the mammal. (A-C) Maps of skin sample sites and culture results for mice dorsally-infected with 1 x 10^4^ *wt* (A), *oppA2tn* (B), or *oppA2c* (C) (n = 5). Inoculation sites are designated with a white asterisk. Dotted circles denote capsule placement for larval feeding. (D) Immunoblot analysis of infected mouse sera against *B*. *burgdorferi* whole cell lysates.

We next devised a protocol to assess the ability of the *oppA2tn* mutant to disseminate by the hematogenous and intracutaneous routes. To assess hematogenous dissemination, we cultured blood, daily, five to seven days post-inoculation, the period of peak spirochetemia [44], and organs harvested at the time of sacrifice (4 weeks). To evaluate cutaneous spread, we cultured skin from thirty-two sites (including inoculation site and pinnae) evenly distributed across the dorsa and ventra (Fig 3A–3C). Blood (Table 4) and distal tissues from *oppA2tn-*infected mice (Table 2 –Skin Sampling Experiment) were culture-negative; as before, *oppA2tn* infection elicited minimal serological response (Fig 3D). In contrast to Pilot Experiment 2 (above), complementation in this experiment was not complete, as hearts from *oppA2c*-infected mice were culture negative. The *wt* and *oppA2c* strains were recovered from the large majority of dorsal and ventral sites (139/160 and 149/160 positive sites, respectively; Fig 3A and 3C), whereas the *oppA2tn* mutant was recovered from significantly fewer sites (58/160, Fig 3B) distributed radially from the inoculation sites (also culture positive). Notably, *oppA2tn* spirochetes were recovered from only a handful of sites on the ventral flanks. Cultures from positive skin sites were darkfield-positive within the same time frame (one week) for all three strains.

**Table 4:** *oppA2tn* positive blood culture data.

Strain	*wt*	*oppA2tn*	*oppA2c*
**Day 5**	4/5	0/5	1/5
**Day 6**	4/5	0/5	3/5
**Day 7**	4/5	0/5	1/5
**Total mice culture positive**	5/5	0/5	5/5

### Lack of OppA2 does not impair spirochete survival in murine blood *ex vivo*

**Fig 4:**
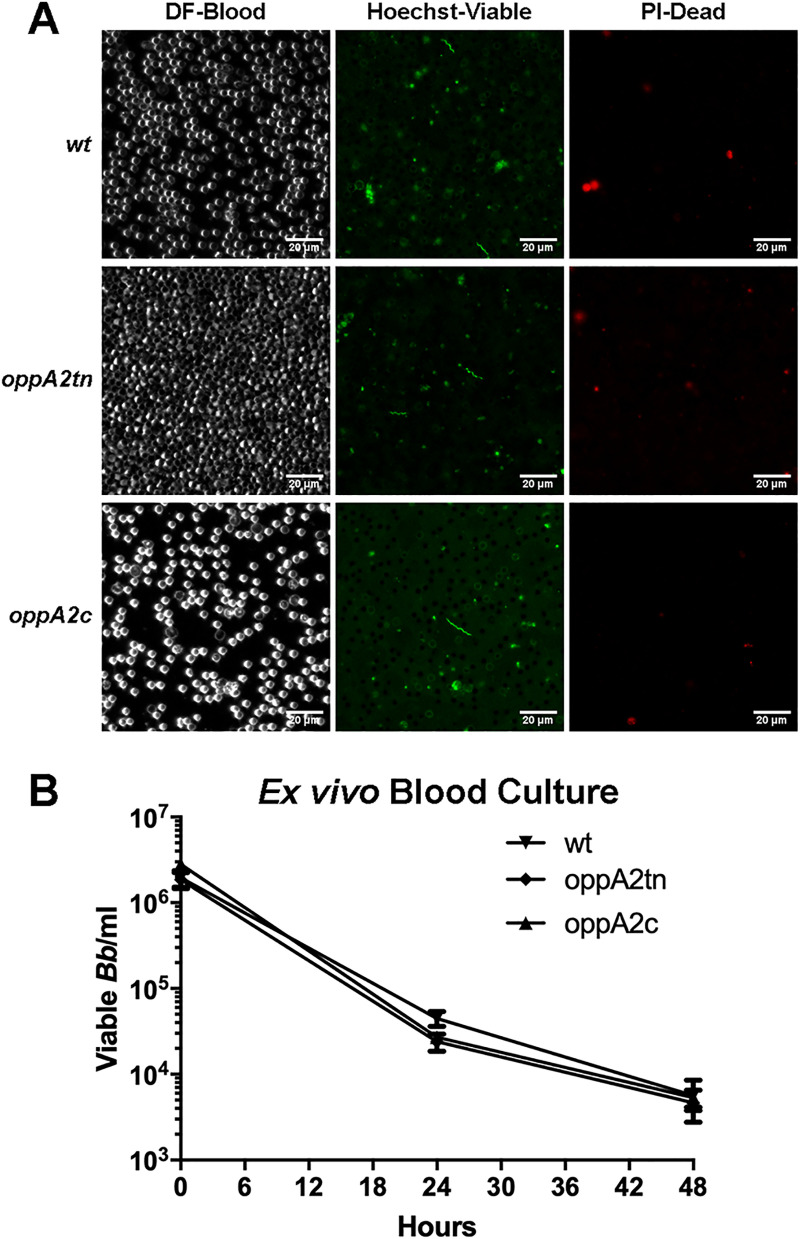
*oppA2tn* survives comparably to *wt* during *ex vivo* blood culture. (A) Darkfield microscopy (DF) and epifluorescence microscopy of Hoechst- (HS—Viable) and propidium iodide-stained (PI–Dead) *wt*, *oppA2tn*, and *oppA2c* spirochetes after 24 hrs ofincubation in whole mouse blood, 400x magnification. (B) Viable spirochetes recovered from blood culture assessed by semi-solid plating at timepoints 0, 24, and 48 hrs.

The above negative blood culture results (Table 4) suggested that *oppA2tn* is either unable to invade the vasculature or survive within the blood compartment. To evaluate the latter possibility, we devised an *ex vivo* assay to evaluate spirochete viability during prolonged incubation in blood. *wt*, *oppA2tn*, and *oppA2c* cultures were washed in PBS and incubated for 48 h in triplicate in undiluted mouse blood at a concetration of 1 x 10^6^ spirochetes/ml. As darkfield microscopy could visualize only red blood cells, at 24 h, spirochete viability was assessed qualitatively by live/dead staining and epifluorescence microscopy (Fig 4A). Microscopy revealed viable spirochetes for each strain which displayed normal cell morphology. At 24 and 48 hrs, samples were collected for semi-solid plating (Fig 4B). None of the strains replicated during culture in blood, and all exhibited essentially identical decreases in viability over time, confirming that *oppA2tn* fared no worse than *wt* and *oppA2c* spirochetes during blood culture.

### *oppA2tn* can survive and be acquired depending on tick placement

**Fig 5:**
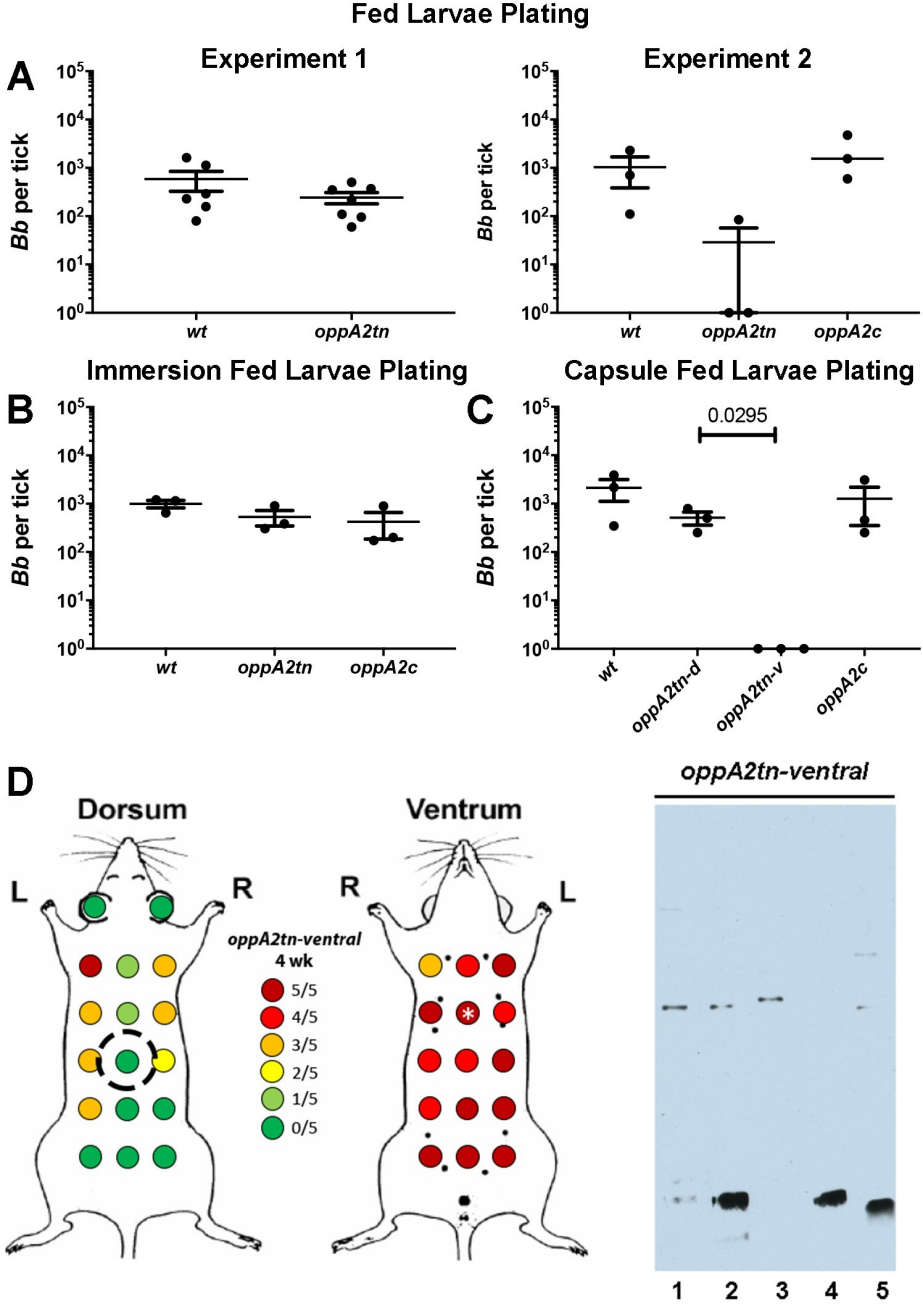
*oppA2* is dispensable during larval acquisition. (A) Two preliminary experiments measuring spirochete burdens (mean ±SEM) assessed by colony counts in midguts from larvae naturally fed on mice infected with *wt*, *oppA2tn*, or *oppA2c*. (B) Colony counts (mean ±SEM) from midguts of larvae immersion fed with *wt*, *oppA2tn*, or *oppA2c* cultures and subsequently fed on naïve C3H/HeJ. (C) Colony counts (mean ±SEM) from midguts of larvae naturally fed on mice infected with *wt-dorsally*, *oppA2tn-dorsally*, *oppA2tn-ventrally*, or *oppA2c-dorsally*. (D) Maps of skin sample sites and culture results and immunoblot analysis using *B*. *burgdorferi* whole cell lysates of sera from mice ventrally-infected with 1 x 10^4^ *oppA2tn* (n = 5). Inoculation sites are designated with a white asterisk. Dotted circle denotes capsule placement for larval feeding.

In two initial experiments, we evaluated the ability of *oppA2tn* to be acquired by and survive in the arthropod vector. In experiment one, larvae fed on mice infected with *wt* or *oppA2tn* contained equivalent spirochete burdens (Fig 5A, Experiment 1, left panel). However, in experiment two, which included *oppA2c*, two of three pools of *oppA2tn*-infected larvae had no viable spirochetes, while spirochete numbers in the third pool were ~ 1 log lower than the *wt* and *oppA2c* pools (Fig 5A, Experiment 2, right panel).

To explain this disparity, we first conducted experiments using the larval immersion technique [43] to evaluate whether the *oppA2tn* mutant could survive within the midgut during feeding; as shown in Figs 5B and S4, the mutant survived the blood meal as well as *wt* and *oppA2c*. The experiments depicted in Fig 5A were done by whole body infestation with feeding larvae randomly distributed on the mouse. We, therefore, considered the possibility that the differences in acquisition in the two preliminary experiments were due to non-uniform distribution of the mutant. To test this, we conducted acquisition experiments by dorsal placement of larvae in feeding capsules. In addition to the three groups of dorsally infected mice described in Fig 3A–3C, we included a fourth cohort of mice inoculated ventrally with *oppA2tn* (Fig 5D). Skin cultures of the ventrally inoculated *oppA2tn* mice at the time of sacrifice (4 weeks; Fig 5D) demonstrated a converse pattern to that obtained following dorsal inoculation; namely, spirochetes reached only the dorsal flanks. Larvae capsule-fed on the dorsally infected mice acquired equivalent bacterial burdens, whereas larvae fed on mice infected ventrally with *oppA2tn* did not acquire spirochetes (Fig 5C). Thus, the mutant does not display an innate acquisition defect; rather, acquisition of the mutant depends upon the placement of ticks in relation to the site of needle-inoculation.

### *oppA2tn* displays defective dissemination via nymphal inoculation

**Fig 6:**
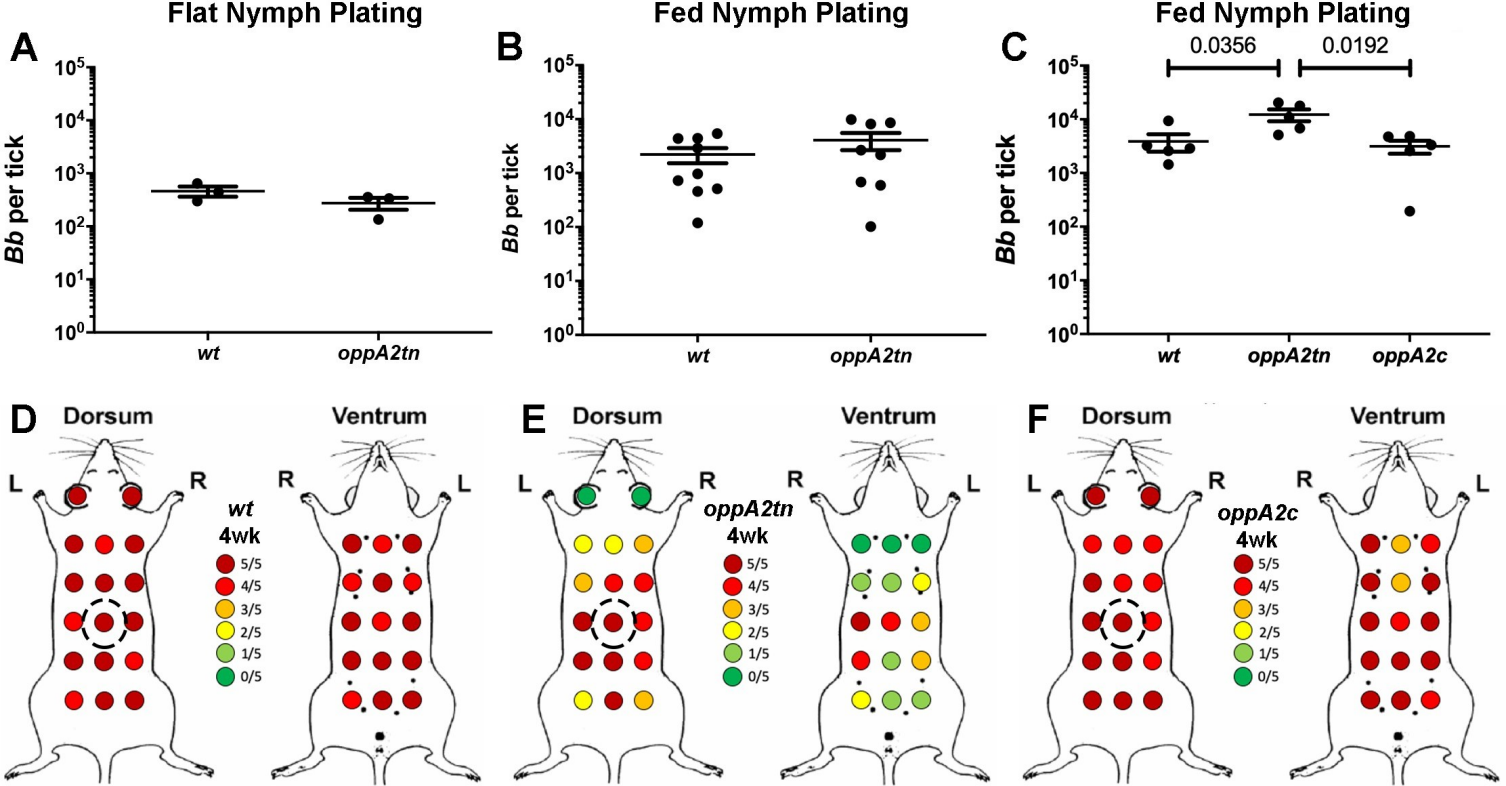
*oppA2* is dispensible for survival in the tick midgut and transmission. (A-B) Colony counts (mean ±SEM) from midguts of (A) flat and (B) fed nymphs infected with *wt* or *oppA2tn*. (C) Colony counts (mean ±SEM) from midguts of fed nymphs infected with *wt*, *oppA2tn*, or *oppA2c*. Each data point represents a separate pool of ticks. Statistical analysis of tick studies was determined by unpaired Student’s *t* test. (D-F) Maps of skin sample sites and culture results for mice dorsally-infected via nymph feeding with *wt* (A), *oppA2tn* (B), or *oppA2c* (C) (n = 5). Dotted circles denote capsule placement for nymphal feeding.

We used the *oppA2tn-*infected larvae from our first natural acquisition experiment (Fig 5A, Experiment 1, left panel) to assess the ability of the mutant to be transmitted and disseminate by nymphal inoculation. Survival of the molt was confirmed by recovery of equivalent numbers of *wt* and *oppA2tn* from flat nymphs by plating (Fig 6A). When *wt-* and *oppA2tn-*infected nymphs were fed to repletion on naïve mice (~15 nymphs/mouse), the resulting colony counts were virtually identical (Fig 6B), further confirming that loss of OppA2 does not affect survival in the tick. At two-weeks post-drop-off, inoculation sites were culture positive in four of the nine *oppA2tn* nymph-infected mice, while samples from all distal tissues were culture negative (Table 2 –Nymphal Transmission Experiment); *oppA2tn* nymph-infected mice also failed to seroconvert (S5 Fig). Samples from *wt* infected mice were consistently culture positive.

We next sought to determine whether tick inoculation with *oppA2tn* fully replicates the dissemination defect observed following infection by needle. Therefore, we inoculated naïve mice with nymphs infected with *wt*, *oppA2tn*, *and oppA2c* and assessed infection by culture of distal sites and extensive skin-sampling four-weeks post-feeding. As expected, colony counts of replete nymphs fed on naïve mice (~15 nymphs/mouse) were virtually identical among the three strains (Fig 6C) confirming, once again, that *oppA2tn* survives normally in feeding ticks. As in prior experiments, pinnae and organs from the *oppA2tn*-infected mice were culture negative (Table 2 –Nymphal Skin Sampling Experiment). With the exception of hearts, distal tissues from *oppA2c* infected mice were culture positive. The *wt* and *oppA2c* strains were recovered from the large majority of dorsal and ventral sites (151/160 and 144/160 positive sites, respectively; Fig 6D and 6F). Though all inoculation sites were culture positive for the *oppA2tn* infected mice, significantly fewer skin samples yielded positive cultures (84/160, Fig 6E). In addition, skin mapping from mice infected with *oppA2tn* displayed a similar radial dissemination pattern their needle-inoculated counterparts (compare Figs 3B and 6E), with slightly increased coverage on the ventral aspects; in contrast, *wt* and *oppA2c* infected mice demonstrated robust cutaneous dissemination (Fig 6D and 6F). The slight increase in recovery from ventral sites of *oppA2tn* infected mice, as compared to needle-inoculation, could be due to the larger surface area of the ‘inoculation site’ with capsule feeding. As with needle-inoculated mice, positive skin cultures for all three strains were darkfield positive within the same time frame (one week).

### *oppA5* is dispensable within the tick but required for persistence within the mouse

**Fig 7:**
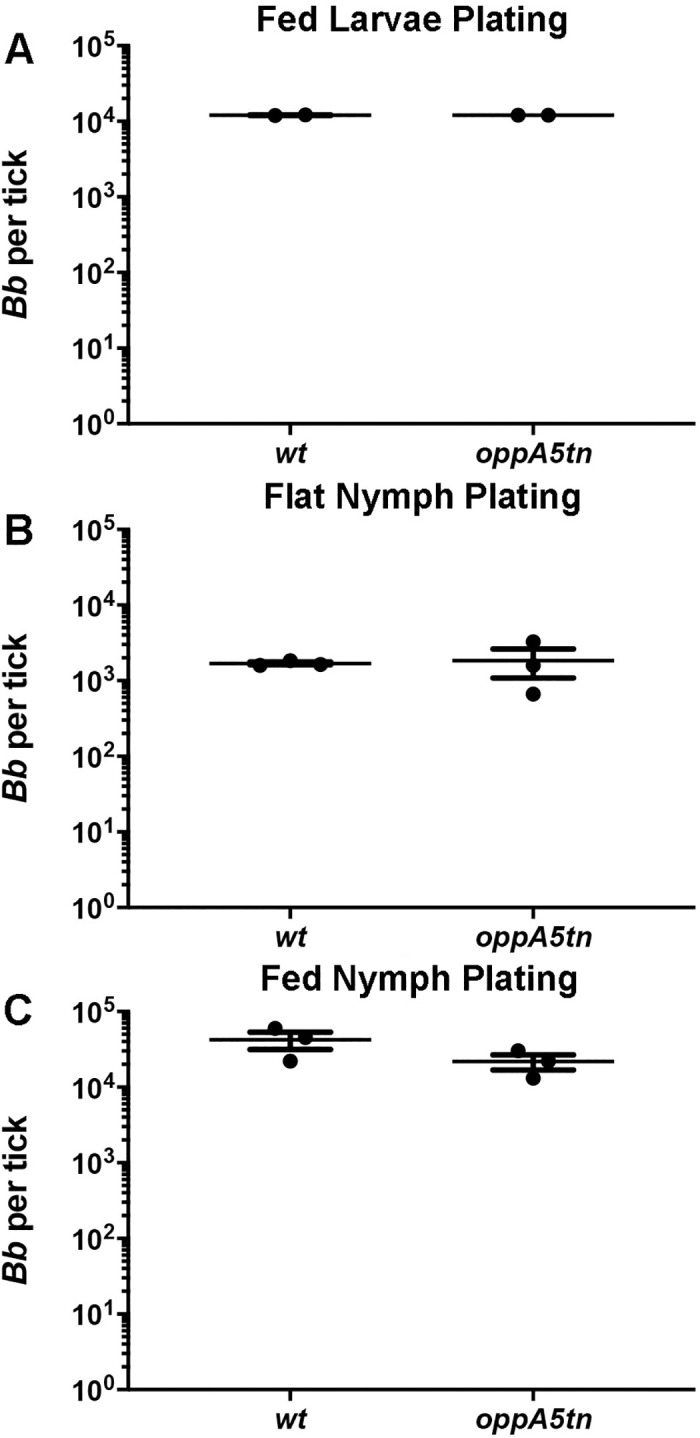
*oppA5* is dispensable in ticks. (A) Colony counts (mean ±SEM) of midguts from larvae immersion fed with *wt* and *oppA5tn*, (B) subsequent molted flat nymphs, and (C) fed nymphs. Each data point represents a separate pool of ticks. Statistical analysis was determined by unpaired Student’s *t* test.

Recently, we demonstrated that an *oppA5tn* mutant displays an impaired ability to persist in needle-inoculated mice which could be restored by complementation [38]. *oppA5*, the only RpoS-regulated Opp component [17,29,32,33,38], is not expressed in feeding nymphs [14], an RpoS-ON state [5], because it is repressed by c-di-GMP [37]. These expression data lead to the prediction that OppA5 is not required for survival within ticks. To confirm this, we assessed the tick phenotype of the *oppA5tn* mutant using immersion feeding to circumvent its attenuation in mice. Spirochete burdens in immersion-fed larvae (Fig 7A) and flat nymphs following the molt (Fig 7B) were essentially identical by plating. Flat nymphs infected with *wt* or *oppA5tn* spirochetes were fed on naïve mice; Fig 7C shows that nymphal burdens at repletion, also by plating, were comparable for the two strains. At four weeks, inoculation sites in all mice were culture positive, confirming transmission. Consistent with the previously described phenotype observed by needle-inoculation [38], the *oppA5tn*-infected mice showed mild attenuation at the four week timepoint (9/12 vs 12/12 positive cultures for *oppA5tn* and *wt*, respectively; Table 5).

**Table 5:** *oppA5tn* positive tissue culture data from nymph fed mice.

Strain	Ear	Inoculation Site	Tibiotarsal Joint	Heart	Total Tissues	Total Mice
**Immersion-infected nymphs—4 weeks PI**						
***wt***	3/3	3/3	3/3	3/3	12/12	3/3
***oppA5tn***	2/3	3/3	1/3	3/3	9/12	3/3

## Discussion

Though *B*. *burgdorferi* cannot utilize AAs as carbon sources [7], they are required for biosynthesis of proteins and peptidoglycan (PG). The absence of AA biosynthetic pathways obligates the spirochete to procure these essential nutrients using a small repertoire of dedicated AA transporters in concert with a complex oligopeptide uptake system [7]. As the singular ATPase is the energetic lynchpin of the Opp system, we previously targeted this component via conditional mutagenesis to demonstrate that peptide uptake is essential for *B*. *burgdorferi* viability, even within an enriched cultivation medium containing a full complement of AAs [14]. Peptide starvation of the ATPase^cond^ mutant *in vitro* resulted in a novel, pleomorphic phenotype characterized by dysregulated cell envelope biogenesis and arrested cell division. ATPase^cond^ spirochetes cultivated in DMCs host-adapted but displayed similar growth defects, providing an *in vivo* morphological explanation for the mutant’s inability to establish a foothold following needle inoculation. It is unclear, however, whether these maladaptive responses are strictly nutritional, the result of AA deprivation and as yet undefined mechanisms for sensing diminished intracellular AA pools [45], or reflective of aberrant environmental sensing [46,47]. Transport of signaling peptides by Opp systems is well described in Gram-positive bacteria [48–51], although there is no evidence to date for peptide-based cell-cell communication in *B*. *burgdorferi*. In the present manuscript, we used *oppA* mutants to confirm that peptide acquisition is essential within the tick as well as the vertebrate reservoir, demonstrating that *B*. *burgdorferi* has evolved a unique ‘brand’ of nutritional virulence spanning its entire lifecycle. We also confirmed the conjecture emerging from our previous studies [14] that *B*. *burgdorferi* employs temporal regulation in concert with structural variation of Opp components to ensure acquisition of sufficient AAs in every biological niche in which it persists. It is noteworthy that none of the *oppA* mutants examined herein exhibited major growth or morphologic defects *in vitro*, underscoring a striking dichotomy between the redundancy of the Opp system *in vitro* and the functional specialization of its components *in vivo*. An unexpected facet of this specialization may be a signaling function linking peptide acquisition to dissemination during the mammalian phase of the life cycle.

**Fig 8:**
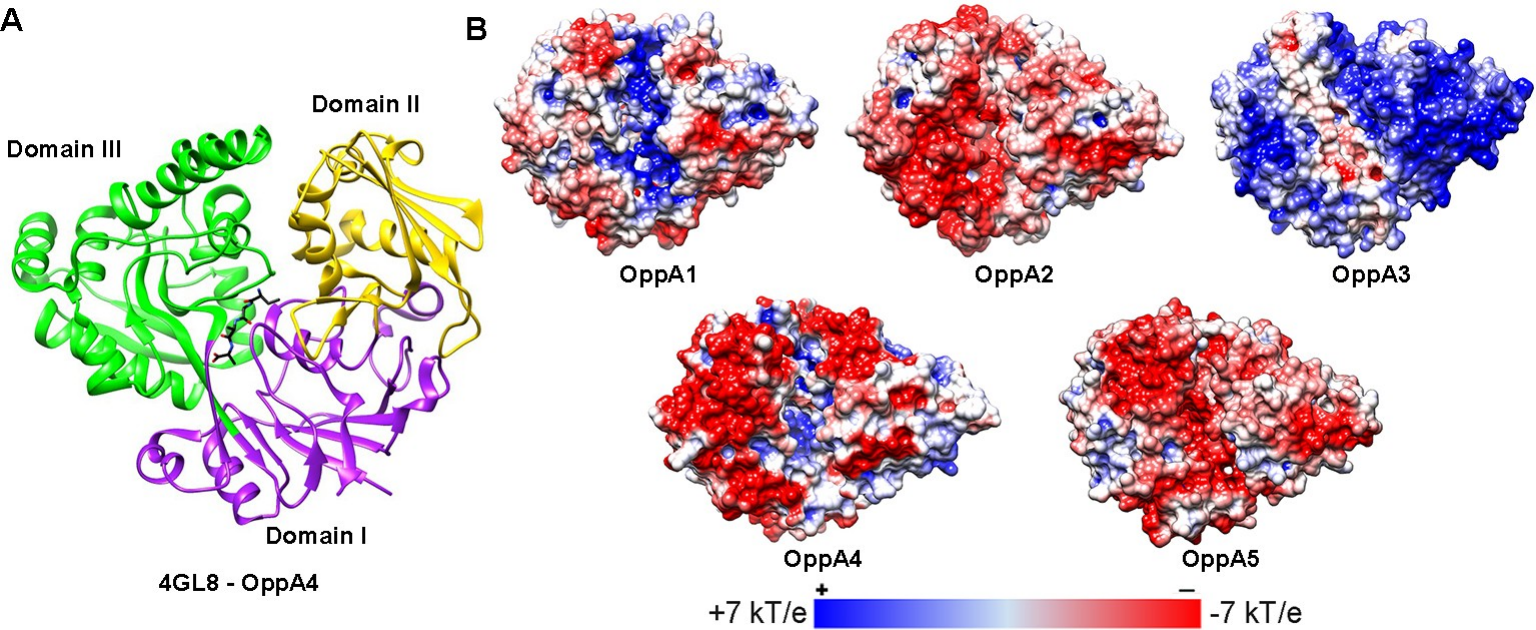
The binding pockets of OppA1-5 demonstrate variations in electrostatic distribution. (A) Ribbon model of OppA4 crystal structure (PDB: 4GL8) [26] showing domains I (purple–hinge region), II (yellow), and III (green) and the Ala_4_ liganded peptide (black). (B)Electrostatic distributions unliganded (open) OppA1-5, modeled against unliganded *E*. *coli* OppA (PDB: 3TCH) [24]. Electrostatic models have been rotated 45° on the z-axis to show the permease-binding regions (region framing the binding site as clearly seen on the positively charged surface of OppA3) or the binding pockets (clearly distinguished as the positively charged pocket along the center of OppA1). Figure was adapted from Groshong *et al*. [14].

The major theme to have emerged from this study is that individual borrelial OppAs appear to function in either the tick or murine environment. Of the five OppAs, OppA1 and OppA2 illustrate a clear dichotomy, with unique transcriptional profiles throughout the cycle and the greatest structural divergence in binding cavity size, electrostatics (Fig 8), and non-synonymous liganding residue diversity [14]. Prior qRT-PCR analysis revealed that transcription of *oppA1* predominates in feeding ticks [14]. Experiments herein using an *oppA1tn* mutant brought to light a striking, tick-specific phenotype associated with this transcriptional profile. While needle-inoculated *oppA1tn* spirochetes established persistent murine infection comparable to *wt B*. *burgdorferi*, semi-solid plating and IFA revealed that the mutant not only lacked viability in ticks but also lysed in larval midguts. qPCR unequivocally ruled out the possibility that the mutant had failed to transit to the vector; furthermore, immersion feeding, which bypasses the infectious phase, yielded an identical phenotype. The PG sacculus maintains the integrity of bacterial cell envelopes against high turgor pressures [49,52–54]. Thus, one plausible explanation for the *oppA1tn* phenotype is that insufficient transport of peptides rich in AAs essential for PG synthesis (*e*.*g*., arginine/ornithine, alanine, glycine, and glutamate [55]) renders the mutant vulnerable to lysis from changes in osmolality within the midgut during the blood meal [56]. Another possibility, which is not mutally exclusive, is that OppA1 imports peptides containing AAs that serve as osmoprotectants (*e*.*g*., proline and glutamate) [18]. Our thinking that the PG sacculus is involved in the *oppA1tn* phenotype is based on its resemblance to the lytic phenotype of *rrp1* and *hk1* mutants during tick feeding [18,37, 57–59]. Rrp1 and Hk1 form a two-component system responsible for generation of c-di-GMP, an important regulator for tick phase genes, including importers of chitobiose and N-acetylglucosamine, carbohydrates required for PG synthesis [11,12,31,58]. While the mechanism(s) underlying the protective function of OppA1 during tick feeding remains to be determined, our results argue that evolution has tailored the binding capacity of OppA1 to the range of peptides available within this unique milieu. The blood meal provides an abundance of proteins not just from serum but also intraluminal lysis of erythrocytes (*e*.*g*., hemoglobin and serum albumin) [60–62]. Furthermore, tick saliva contains a wide array of small peptides, which, upon re-ingestion, provide an arthropod-specific source of peptides [63]. HtrA, a *B*. *burgdorferi* surface protease [64–66], and/or host-derived proteases such as plasminogen bound to the borrelial surface [67] also may contribute to protein degradation within the midgut.

In contrast to relapsing fever spirochetes, which are acquired by their soft tick and louse vectors from blood, *Ixodes spp*. acquire LD spirochetes from the dermis [68–70]. Thus, maintenance of the *B*. *burgdorferi* enzootic cycle depends upon widespread dissemination of spirochetes within the reservoir host, often thought to occur predominantly via hematogenous spread [44,71–74]. However, two lines of evidence argue for the importance of intracutaneous migration: (i) bioluminescence imaging of infected laboratory mice shows extensive lateral spread at early timepoints (i.e., within two weeks) [75–77], and (ii) the hallmark cutaneous lesion of LD in humans, erythema migrans, which can be expansive, is the result of lateral migration of spirochetes [78–81]. One might surmise, therefore, that extensive cutaneous dissemination also occurs within the reservoir host. Using a Tn-seq format in which tissue samples were pooled, Troy *et al*. [40] previously showed that an *oppA2tn* mutant was significantly attenuated. An unexpected outcome from our study is the discovery that the loss of OppA2, a protein with a presumptive nutritive function, results in a striking defect in dissemination. Our finding that *oppA2tn* retained viability at *wt* levels in our *ex vivo* blood assay argues that the failure of the mutant to disseminate hematogenously was due to an inability to access the vascular compartment. Although spirochetes lacking OppA2 could not spread hematogenously, they could migrate intracutaneously from the inoculation site, eventually gaining access to approximately 50% of the mouse’s surface area. Notably, the *oppA2tn* mutant was never recovered from pinnae, a site widely accepted as an indicator of hematogenous dissemination [71]. The *oppA2tn* phenotype, therefore, provides the first genetic evidence that efficient and complete dissemination of *B*. *burgdorferi* throughout the skin requires a combination of intracutaneous migration from the site of inoculation and hematogenous seeding of distal skin. Further experiments tracking spirochete migration in skin and visualizing individual organisms *in situ* will be needed to determine whether (and, if so, the extent to which) impaired intracutaneous migration contributes to the diminished spread of *oppA2tn* in skin. Complementation confirmed that the dissemination defect was due to loss of *oppA2*, although infection of hearts was not fully restored. *oppA2* expression *in vivo* is a combined result of transcription via the operon’s *oppA1* promoter and an internal *oppA2* promoter [14,17,19]. The partial complementation observed with *oppA2c* likely reflects sub-optimal expression of *oppA2* from just the *oppA1* promoter in the mammalian environment (Fig 1A).

Unexpectedly, we could not definitively demonstrate a nutritive function for OppA2 *in vitro* or *in vivo*. While the mutant displayed a modest growth defect during DMC cultivation, it maintained *wt* burdens and viability in the skin at least four weeks post-inoculation and survived as well as *wt* during *ex vivo* blood cultivation. Therefore, if OppA2 does contribute to nutrient acquistion, *oppA2tn* presumably can compensate for its loss via its other OppAs and/or limited reportoire of free AA transporters. In any event, it seems difficult to attribute the mutant’s dissemination defectly solely to AA deficiency. Indeed, our collective results lead us to consider the intriguing possiblity that OppA2 also functions in a signaling capacity during the mammalian phase of the life cycle. We can envision two mechanisms by which this might occur. One is that peptides (or their degradation products) imported by OppA2 activate an as yet unidentified regulatory pathway [82] that orchestrates expression of adhesins and other molecules *B*. *burgdorferi* needs to access and penetrate dermal vasculature [83]. Conceivably, this pathway would be part of the program for mammalian host adaptation *B*. *burgdorferi* undergoes that is not RpoS-mediated. Another is that the inability of *oppA2tn* to transit from the dermis to the dermal vasculature reflects impaired chemotaxis. Studies in *E*. *coli* provide a clear precedent as well as a mechanism for peptide-mediated chemotaxis. *E*. *coli* mutants in either the methyl-accepting chemotaxis protein, Tap, or the dipeptide SBP, DppA, fail to chemotax toward dipeptides. The accepted interpretation of these results is that liganded DppA transduces a chemotactic signal via interaction with Tap [84–86]. In the case of *B*. *burgdorferi*, liganded OppA2 could interact with one of its five annotated MCPs. In light of the promiscuous nature of OppA-peptide interactions, OppA2 would then serve as part of a ‘generalized’ sensor of mammalian phase chemoattractant peptides originating from the blood. To date, chemotaxis of *B*. *burgdorferi* towards a number of stimuli has been demonstrated [87–90], though chemotactic responses toward peptides have not been evaluated. Whereas *B*. *burgdorferi* motility mutants are cleared from the inoculation site within several days of infection [91–94], *oppA2tn* survives for weeks in the skin, a major immune organ [95], inducing only a negligible antibody response. To the best of our knowledge, this is the first report of long term survival of *B*. *burgdorferi* without immune recognition.

The reservoir competence of a mammalian host for LD spirochetes is defined by its tolerance of the bacterium [96–98]. However, in order to exploit this tolerance, *B*. *burgdorferi* must employ a complex parasitic strategy that involves virulence determinants [4,5,73,83,99], immune evasion mechanisms [100–103], and usurpation of the host’s metabolic output [6–9,11–13]. Previous transcriptional analyses [14] led to the prediction that OppA2 and OppA5 function primarily within the mammal and, along with our prior study [38], we now have evidence that these two OppAs are, indeed, indispensable for widespread and persistent murine infection. Moreover, their dispensability within the tick furthers our concept of host-specific compartmentalization of OppA function. Instead of working in tandem, however, OppA2 and OppA5 appear to function in a hierarchical manner: OppA5’s ability to promote persistence relies upon OppA2’s ability to facilitate dissemination. Although the conformations of the OppA2 and OppA5 binding pockets are similar, differences in electrostatics (Fig 8) and residues lining the pocket [14] suggest divergent peptide repertoires. A major unsolved question is the source of peptides during infection, especially when one considers that the mammalian reservoir is a non-inflammatory milieu [104]. Regardless, in comparison with the feeding tick, the mammal is a peptide-limited environment. Conceivably, peptide limitation establishes a ‘set’ point for borrelial replication within the mammal, serving as a contributing determinant of the paucibacillary nature of *B*. *burgdorferi* infection [96,105,106].

## Methods

### Ethics statement

All animal experiments were performed in strict accordance with protocols approved by the UConn Health Center Institutional Animal Care and Use Committee (Animal Welfare Assurance No. A347-01) and in compliance with the Guide for the Care and Use of Laboratory Animals of the National Institutes of Health.

### Bacterial strains and culture conditions

All strains used in this study are listed in S1 Table. TOP10 or Stellar *Escherichia coli* strains were cultured in Luria-Bertani (LB) broth or on LB plates with appropriate antibiotics (kanamycin [Kan; 50 μg/ml], gentamycin [Gent; 10 μg/ml], ampicillin [Amp; 100 μg/ml], and spectinomycin [Spec; 100 μg/ml]) at 37°C. All *Borrelia burgdorferi* strains used in this study are derivatives of B31 5A18 NP1 [41,107]. *B*. *burgdorferi* strains were cultivated in modified Barbour-Stoenner-Kelly-II (BSK-II) medium [108] supplemented with 6% rabbit serum and appropriate antibiotics (kanamycin [Kan; 400 μg/ml], gentamycin [Gent; 50 μg/ml], erythromycin [Erm; 0.06 μg/ml], and streptomycin [Strep; 100 μg/ml]). Tissues and blood were cultured in BSK-II containing *Borrelia* antibiotic cocktail (BAC; 0.05 mg/ml sulfamethoxazole, 0.02 ml/ml phosphomycin, 0.05 mg/ml rifampicin, 0.01 mg/ml trimethoprim, and 2.5 μg/ml amphotericin B). Temperature-shift experiments were carried out as previously described [109]. Plasmid content of *B*. *burgdorferi* strains was determined using the multiplex approach as described in Bunikis *et al*. (S6 Fig) [110]

### Generation of mutant and complement strains

#### oppA2tn

The *oppA2tn* mutant was reconstructed by amplification of the *bb0328-9* region from the original *oppA2tn* mutant (T06TC269) with ~1 kb of DNA flanking the *tn* insertion. As the *tn* insertion includes an *E*. *coli* origin of replication, the amplified fragment was self-ligated to generate pEcAG233 (Fig 1A), which then was transformed into wild-type B31 5A18 NP1. Subsequent clones were confirmed by PCR to carry the *tn* insertion in *oppA2* and screened for plasmid content (S6 Fig) as described above.

#### oppA1c

The coding region for *oppA1* was amplified along with the upstream 500 bp to include the *oppA1* promoter (P*oppA1*) flanked by AatII restriction enzyme sites. We modified a cp26 crossover vector containing *aacC* and P*flaB-GFP* (pMC2498) [37] in order to utilize the cp26 site for complementation. The antibiotic marker and *gfp* cassette were removed by inverse PCR and self-ligation via engineered AatII sites (pEcAG265). The *aadA* marker [111] was introduced into the MCS via a BamHI-restriction site (pEcAG326) The complementation fragment was cloned into the AatII site of pEcAG326 to generate pEcAG341 (Fig 1A). The plasmid was confirmed by sequencing and transformed into the *oppA1tn* mutant. Subsequent clones were screened by PCR for wild-type *oppA1* and plasmid content (S6 Fig).

#### oppA2c

The coding region for *oppA2* was amplified and fused with the 500 bp P*oppA1* with AatII sites flanking the P*oppA1*-*oppA2* fragment. The complementation fragment was cloned into the AatII site of the cp26 crossover vector pEcAG326 to generate pEcAG342 (Fig 1A). The plasmid was confirmed by sequencing and transformed into the *oppA2tn* mutant. Subsequent clones were screened by PCR for wild-type *oppA2* and plasmid content (S6 Fig).

### Growth curves

*B*. *burgdorferi* strains were inoculated into BSK-II containing appropriate antibiotics at 1 x 10^3^ spirochetes/ml and incubated for up to 6 days at 37°C. Spirochetes were enumerated daily by dark-field microscopy as previously described [14]; all experiments were performed in triplicate.

### SDS-PAGE and immunoblot analysis

Temperature-shifted spirochetes were collected for whole-cell lysates, prepared with Invitrogen NuPage LDS Sample Buffer (ThermoFisher Scientific, Waltham, MA) and boiled. Approximately 2 x 10^7^ spirochetes were loaded in each well of a 12.5% separating polyacrylamide gel. Protein bands were visualized by silver stain, as previously described [29], or transferred to reinforced nitrocellulose (GE Healthcare Life Sciences, Pittsburgh, PA). Rabbit polyclonal antiserum against RpoS [112] (1:1000) was generously provided by Jon Skare (Texas A&M University). Generation of rat polyclonal antisera to OspC [113] (1:12,000) and FlaB [28] (1:6,000) was described previously. Goat anti-rat horseradish peroxidase-conjugated secondary antibody (Southern Biotechnology Associates, Birmingham, AL) at 1:20,000. Antibody responses were assessed using sera from infected mice (1,1000) and a horseradish peroxidase-conjugated goat anti-mouse secondary antibody (Southern Biotechnology Associates, Birmingham, AL) at 1:20,000. Immunoblots were developed using the SuperSignal West Pico chemiluminescence substrate (Pierce, Rockford, IL)

### qRT-PCR

Primers used for qRT-PCR assays are listed in S2 Table. Total RNA was isolated as previously described [114] from triplicate cultures of temperature-shifted spirochetes at late logarithmic phase of growth. cDNAs were generated with and without reverse transcriptase using the SuperScript III First Stand Synthesis System (ThermoFisher Scientific). qRT-PCR assays were developed to measure *oppA1* and *oppA2* transcripts in the *tn* mutants by flanking the transposon insertion site in each gene (Fig 1A). *oppA* transcripts were quantified using SsoAdvanced Universal SYBR Mix (Bio-Rad, Hercules, CA) and normalized to *flaB* transcripts [115] (S2 Table) using SsoAdvanced Universal Probe Mix (Bio-Rad). All assays were performed in quadruplicate with three biological replicates. Generation of internal standards for each assay were previously described [14].

### Generation of host-adapted spirochetes in DMCs

Mammalian-host-adapted spirochetes were generated by cultivation in DMCs as previously described [116,117]. Briefly, *B*. *burgdorferi* strains were grown to mid-logarithmic phase and diluted to 1 x 10^4^ spirochetes/ml. SpectraPor dialysis membrane tubing with a 6–8 kDa cutoff (ThermoFisher Scientific) containing 10 ml of diluted spirochetes were implanted into the peritoneal cavities of female Sprague-Dawley rats (175–200 g). DMCs were explanted after two weeks; spirochetes then were enumerated via dark-field microscopy and evaluated for host adaptation by silver stain.

### *In vitro* blood culture assay

Mouse blood was collected by heart stick and clotting was prevented by the addition of 0.1M sodium citrate. 100ul blood was aliquoted into a 96-well tissue culture plate and 1 x 10^6^ spirochetes/ml were added to each well. The input volume was plated in triplicate for each strain to determine the number of viable spirochetes at timepoint 0 hrs. Plates were incubated at 37C at 5% CO2 and samples were collected at 24 hrs for microscopic evaluation. Samples were incubated for 10 min at room temperature with Hoechst (10μg/ml) and propidium iodide (20μg/ml) for live/dead staining, respectively. Spirochetes were visualized on an Olympus BX41 microscope with a Retiga Exi camera (QImaging, Surrey, BC, Canada). Images were acquired with a 40x (1.4NA) objective and QCapture software v. 2.1 (QImaging). For epifluorescence, an X-Cite Xylis light source and a DAPI HYB filter or HQ:Rdil filter were used to detect Hoechst and propidium iodide, respectively. Images were processed using ImageJ v 1.8.0; and Hoechst and propidium iodide images were colorized green and red, respectively. Samples also were collected at 24 and 48 hrs for semi-solid plating and colony counts.

### Infection studies

Five-to-eight-week-old female C3H/HeJ mice (Jackson Laboratories, Bar Harbor, ME) were used for all infections. Mice were injected intradermally with 1 x 10^4^ spirochetes in a central dorsal location, with the exception of the *oppA2tn* mutant infections that included an additional cohort inoculated ventrally. Between two and five weeks post-inoculation, tissues were collected for culture as described above; mouse sera were collected for Western blots as described above. To evaluate hematogenous dissemination, blood was collected for culture at 5, 6, and 7 days post-inoculation (peak spirochetemia) [44] and 10 μl was inoculated into 5 ml BSK-II with BAC. To measure cutaneous dissemination, thirty-two skin samples, including the inoculation site, were collected as shown in the sample maps (Figs 3A–3C, 5D and 6D–6F) and cultured as described above.

### Tick studies

#### Larval acquisition

At two weeks post-infection, naïve mice were used as a blood meal for pathogen-free *Ixodes scapularis* larvae (Oklahoma State University, Stillwater, OK). Larvae were allowed to feed either by whole body infestation or by feeding within a capsule mounted on the dorsal side of the mouse [114]. At repletion, pools of ten larvae from each mouse were evaluated by semi-solid plating for colonies [118] and qPCR using a 1:10 dilution of DNA and the Taq-man *flaB* assay [115] (S2 Table) for quantification of DNA burdens [119]. Immunofluorescence microscopy of crushed ticks was performed as previously described [114] using KPL anti-*Bb* FITC-conjugated antibody (1:400; SeraCare Life Sciences, Milford, MA). Spirochetes were visualized on an Olympus BX41 microscope with a Retiga Exi camera (QImaging, Surrey, BC, Canada). Images were acquired with a 40x (1.4NA) oil immersion objective and QCapture software v. 2.1 (QImaging). Images were processed using ImageJ v. 1.8.0.

#### Immersion fed larvae

Immersion fed larvae were generated as previously described [43]. Briefly, ~200 naïve larvae were immersed in 2 x 10^8^ spirochetes/ml for 1 hr and washed with PBS. Larvae were allowed to recover overnight in an environmental incubator and then fed on naïve mice by the capsule feeding method [114]. At repletion, triplicate pools of ten larvae were evaluated for spirochete burdens as detailed above.

#### Flat nymphs

Replete larvae were allowed to molt (~3–6 months post-repletion), and triplicate pools of ten molted, unfed nymphs were evaluated for spirochete burdens as noted above.

#### Fed nymphs

Fifteen infected nymphs were fed via the capsule feeding method [114] on the dorsum, at repletion nymphs were split into multiple pools of 3–5 nymphs per mouse and evaluated for spirochete burdens as detailed above. At two weeks post-feeding, mice were evaluated for infection as detailed above for tissue culture and skin sampling.

### Statistics

All statistical *analysis* was performed using Prism 8 (GraphPad, Software, Inc.) with an unpaired Student’s *t* test with two-tailed *p* values and a 95% confidence interval, and data are presented as mean ± standard error of the mean (SEM). *p* < 0.05 was considered statistically significant.

## Supporting information

S1 Fig The *oppA1* and *oppA2 tn* mutants display normal *in vitro* growth and temperature-shift. (A) Darkfield microscopy of *wt*, *oppA1tn*, and *oppA2tn*, 1000x magnification, scale bar represents 20 μm. (B) Growth curves of *wt*, *oppA1tn*, and *oppA2tn* from a starting density of 1 x 10^3^ spirochetes/ml at 37°C (n = 3). (B) Immunoblots of temperature-shifted *wt*, *oppA1tn*, and *oppA2tn* demonstrating equivalent production of RpoS and OspC. *B*. *burgdorferi* cell lysates were standardized using FlaB; molecular weight markers are noted in kDa.
(TIF)

S2 Fig *oppA1* is essential in ticks. (A) Immunoblot analysis using *Bb* whole cell lysates of sera from mice four-weeks post needle-inoculation with 1 x 10^4^ *wt* or *oppA1tn*. Colony counts (mean ±SEM) for spirochetes from midguts of (B) larvae naturally fed on mice infected with *wt* or *oppA1tn Bb* and (C) flat nymphs infected with *wt* or *oppA1tn Bb*. Each data point represents a separate pool of ticks. Statistical analysis was evaluated by unpaired Student’s *t* test.
(TIF)

S3 Fig Infection with the *oppA2tn* mutant elicits a weak serologic response. (A) Immunoblot analysis using *B*. *burgdorferi* whole cell lysates of sera from mice four-weeks post needle-inoculation with 1 x 10^4^ *wt* or *oppA2tn*. (B) Immunoblot analysis using *B*. *burgdorferi* whole cell lysates of sera from mice four-weeks post needle-inoculation with 1 x 10^4^ *wt*, *oppA2tn*, and *oppA2c*. (C) qPCR analysis of *Bb* DNA burdens from inoculation sites at four weeks post-inoculation for needle inoculated mice. (D) SDS-PAGE and silver staining of *wt*, *oppA2tn*, and *oppA2c* cultivated in DMCs. *In vitro* samples of room-temperature (RT) and 37°C to demonstrate expression of OspA and OspC is shown to the left. Molecular weight markers are noted in kDa.
(TIF)

S4 Fig IFA of midguts from replete larvae immersion fed with *wt*, *oppA2tn*, or *oppA2c*. (TIF)

S5 Fig Immunoblot analysis using *Bb* whole cell lysates of sera from mice two-weeks post-inoculation with nymphs harboring *wt* or *oppA2tn*. (TIF)

S6 Fig Plasmid content of strains in this study. (A) Schematic of multiplex-plasmid content for all B31 plasmids. (B) Plasmid contents for all strains used in this study. B31 5A18 NP1 (*wt*) is missing lp56 and lp28-4, as previously published, as are all subsequent strains. *oppA5tn* has lost lp5, a commonly lost plasmid with no known effects on mammalian or tick infectivity. *oppA1c* is missing lp21, which is not required for mouse infectivity [120]. Little is known about the requirement for lp21 during tick infection, though it appears to be unnecessary due to *wt* levels of tick colonization by *oppA1c*.
(TIF)

S1 Table Strains and plasmids used in this study. (TIF)

S2 Table Oligonucleotides used in this study. (TIF)

S1 Movie Basic motility of *wt* spirochetes by darkfield microscopy. (MP4)

S2 Movie Basic motility of *oppA2tn* spirochetes by darkfield microscopy. (MP4)

S3 Movie Basic motility of *oppA2c* spirochetes by darkfield microscopy. (MP4)
