# Supplementary figures and images for: Host-specific functional compartmentalization within the oligopeptide transporter during the Borrelia burgdorferi enzootic cycle
Source: PLoS Pathog. 2021 Jan 11;17(1):e1009180. doi: 10.1371/journal.ppat.1009180 (PMC7822543; doi:10.1371/journal.ppat.1009180)

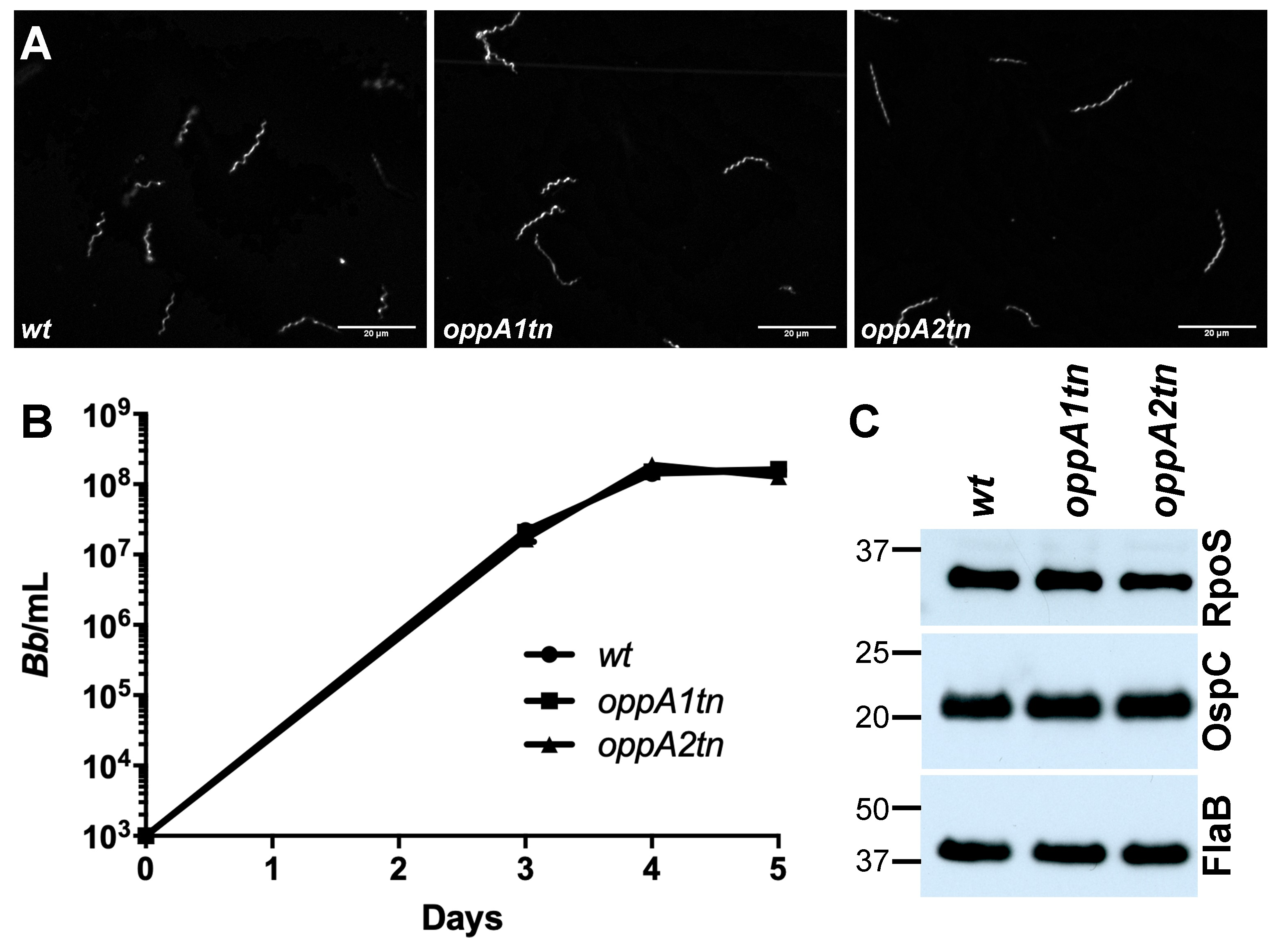

Supplement: S1 Fig — (A) Darkfield microscopy of wt, oppA1tn, and oppA2tn, 1000x magnification, scale bar represents 20 μm. (B) Growth curves of wt, oppA1tn, and oppA2tn from a starting density of 1 x 10^3^ spirochetes/ml at 37°C (n = 3). (B) Immunoblots of temperature-shifted wt, oppA1tn, and oppA2tn demonstrating equivalent production of RpoS and OspC. B. burgdorferi cell lysates were standardized using FlaB; molecular weight markers are noted in kDa. (TIF) [file ppat.1009180.s001.tif]

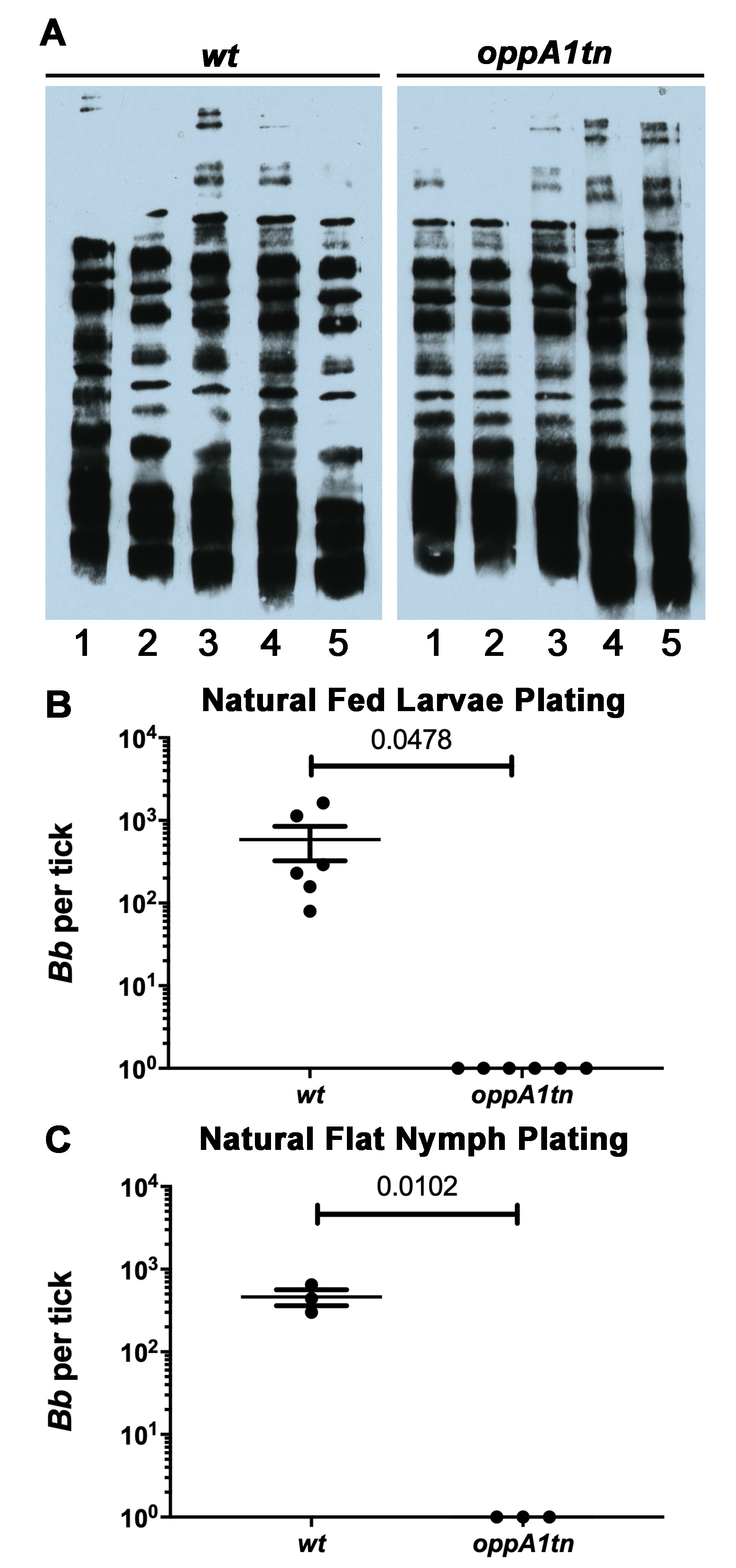

Supplement: S2 Fig — (A) Immunoblot analysis using Bb whole cell lysates of sera from mice four-weeks post needle-inoculation with 1 x 10^4^ wt or oppA1tn. Colony counts (mean ±SEM) for spirochetes from midguts of (B) larvae naturally fed on mice infected with wt or oppA1tn Bb and (C) flat nymphs infected with wt or oppA1tn Bb. Each data point represents a separate pool of ticks. Statistical analysis was evaluated by unpaired Student’s t test. (TIF) [file ppat.1009180.s002.tif]

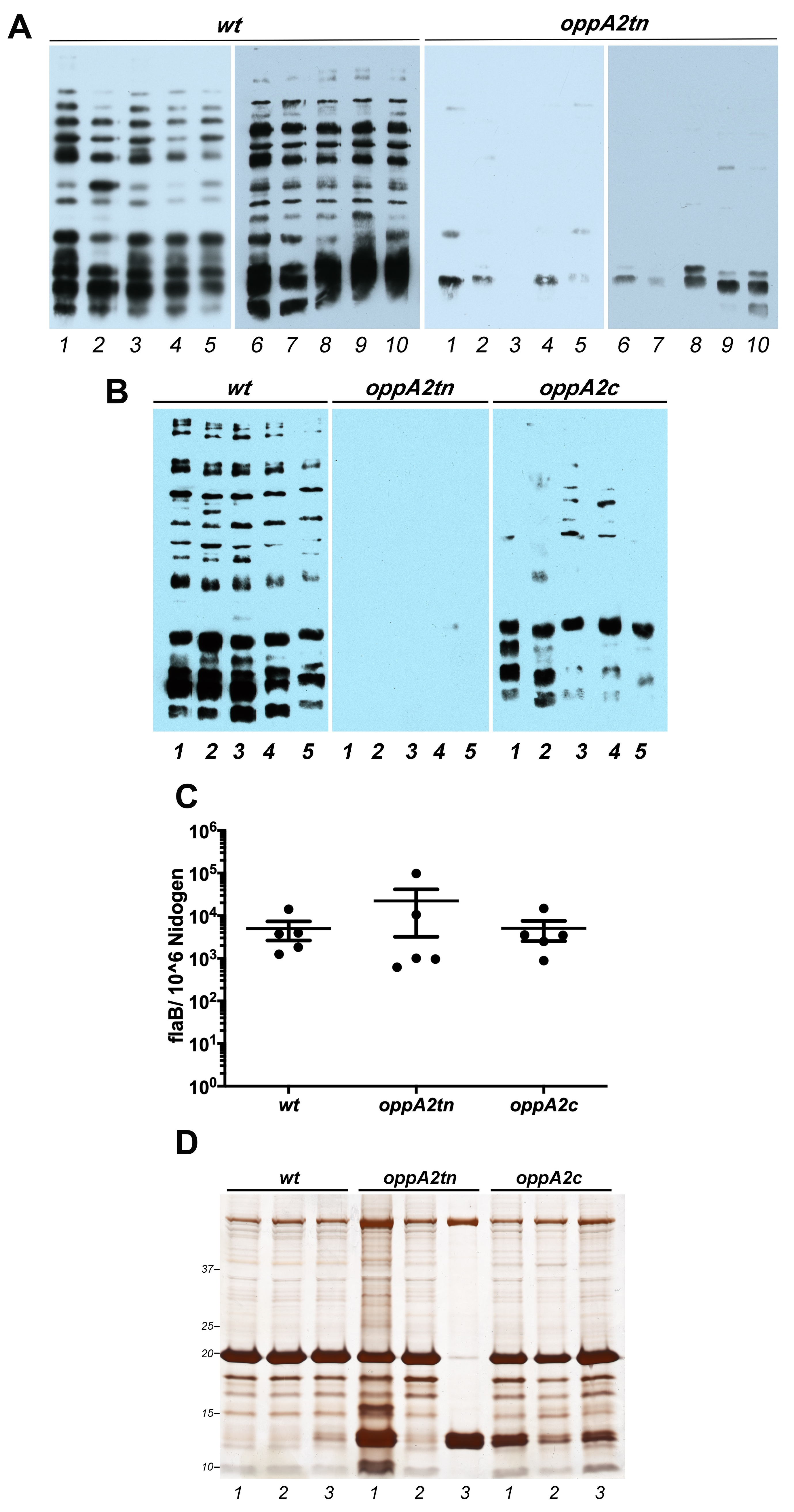

Supplement: S3 Fig — (A) Immunoblot analysis using B. burgdorferi whole cell lysates of sera from mice four-weeks post needle-inoculation with 1 x 10^4^ wt or oppA2tn. (B) Immunoblot analysis using B. burgdorferi whole cell lysates of sera from mice four-weeks post needle-inoculation with 1 x 10^4^ wt, oppA2tn, and oppA2c. (C) qPCR analysis of Bb DNA burdens from inoculation sites at four weeks post-inoculation for needle inoculated mice. (D) SDS-PAGE and silver staining of wt, oppA2tn, and oppA2c cultivated in DMCs. In vitro samples of room-temperature (RT) and 37°C to demonstrate expression of OspA and OspC is shown to the left. Molecular weight markers are noted in kDa. (TIF) [file ppat.1009180.s003.tif]

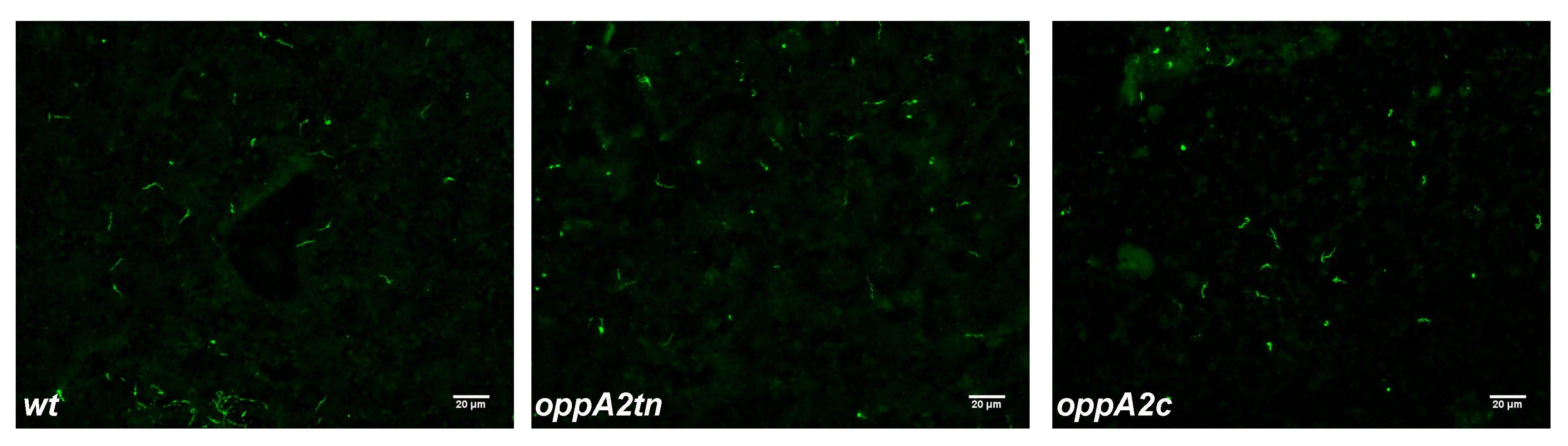

Supplement: S4 Fig — (TIF) [file ppat.1009180.s004.tif]

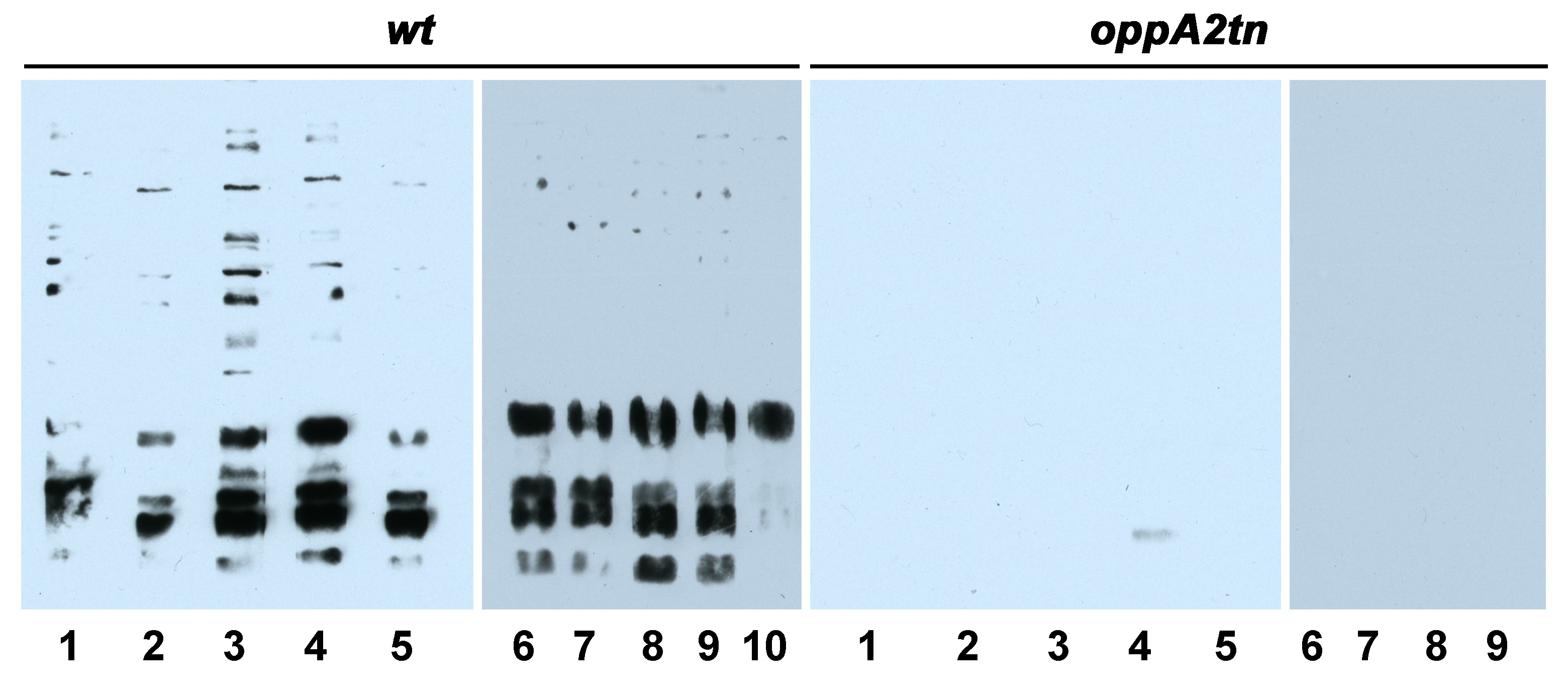

Supplement: S5 Fig — (TIF) [file ppat.1009180.s005.tif]

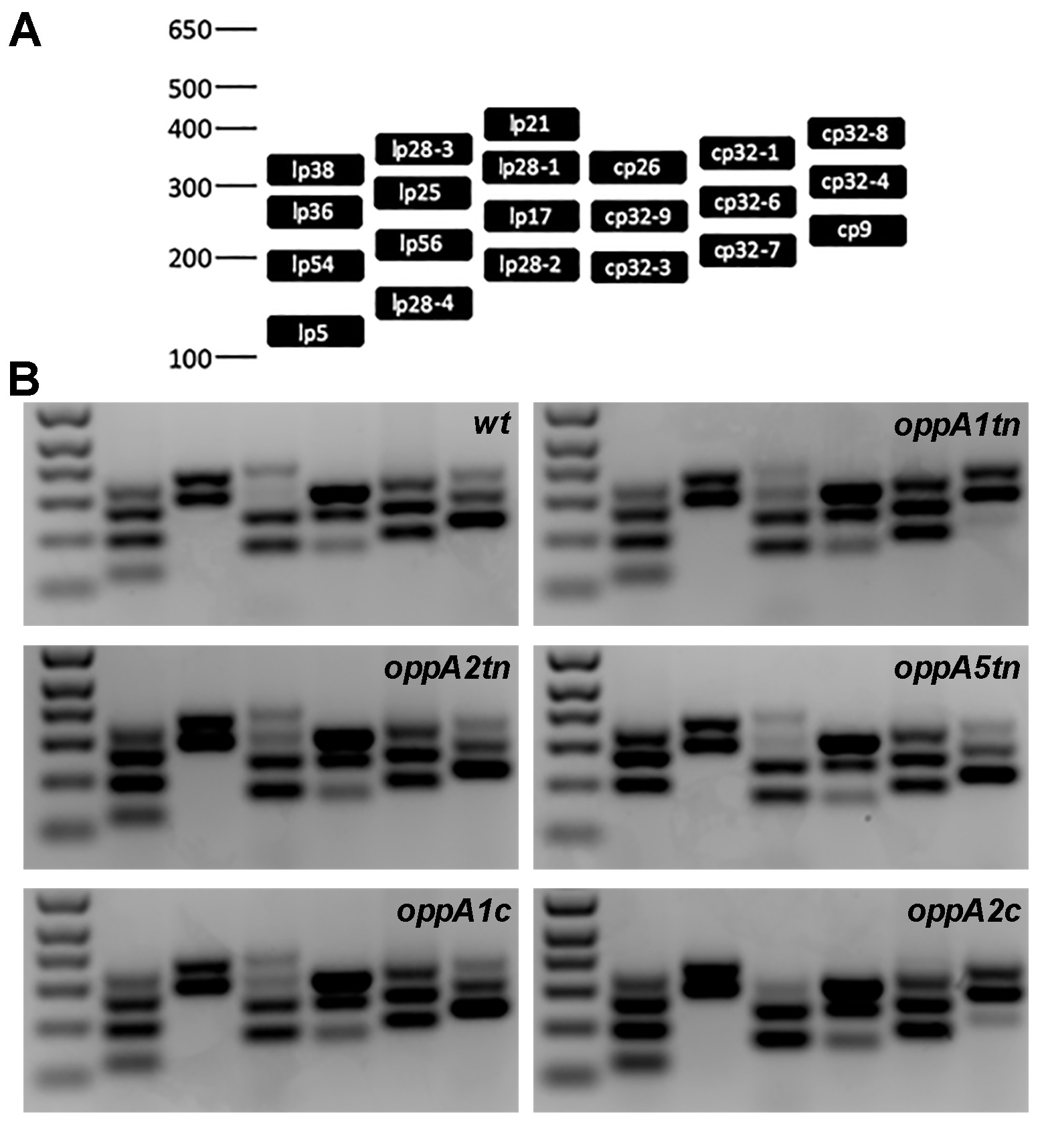

Supplement: S6 Fig — (A) Schematic of multiplex-plasmid content for all B31 plasmids. (B) Plasmid contents for all strains used in this study. B31 5A18 NP1 (wt) is missing lp56 and lp28-4, as previously published, as are all subsequent strains. oppA5tn has lost lp5, a commonly lost plasmid with no known effects on mammalian or tick infectivity. oppA1c is missing lp21, which is not required for mouse infectivity [120]. Little is known about the requirement for lp21 during tick infection, though it appears to be unnecessary due to wt levels of tick colonization by oppA1c. (TIF) [file ppat.1009180.s006.tif]

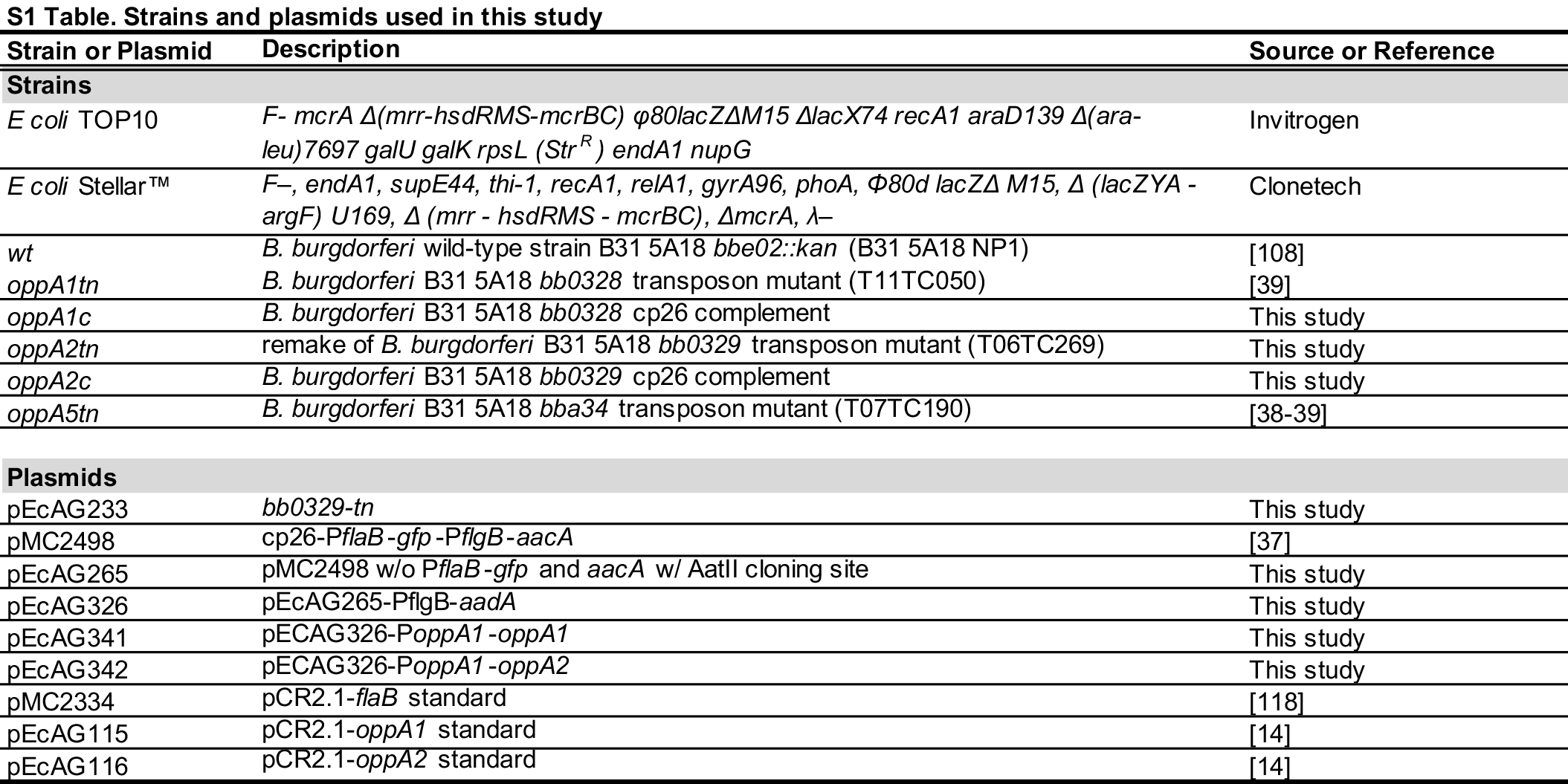

Supplement: S1 Table — (TIF) [file ppat.1009180.s007.tif]

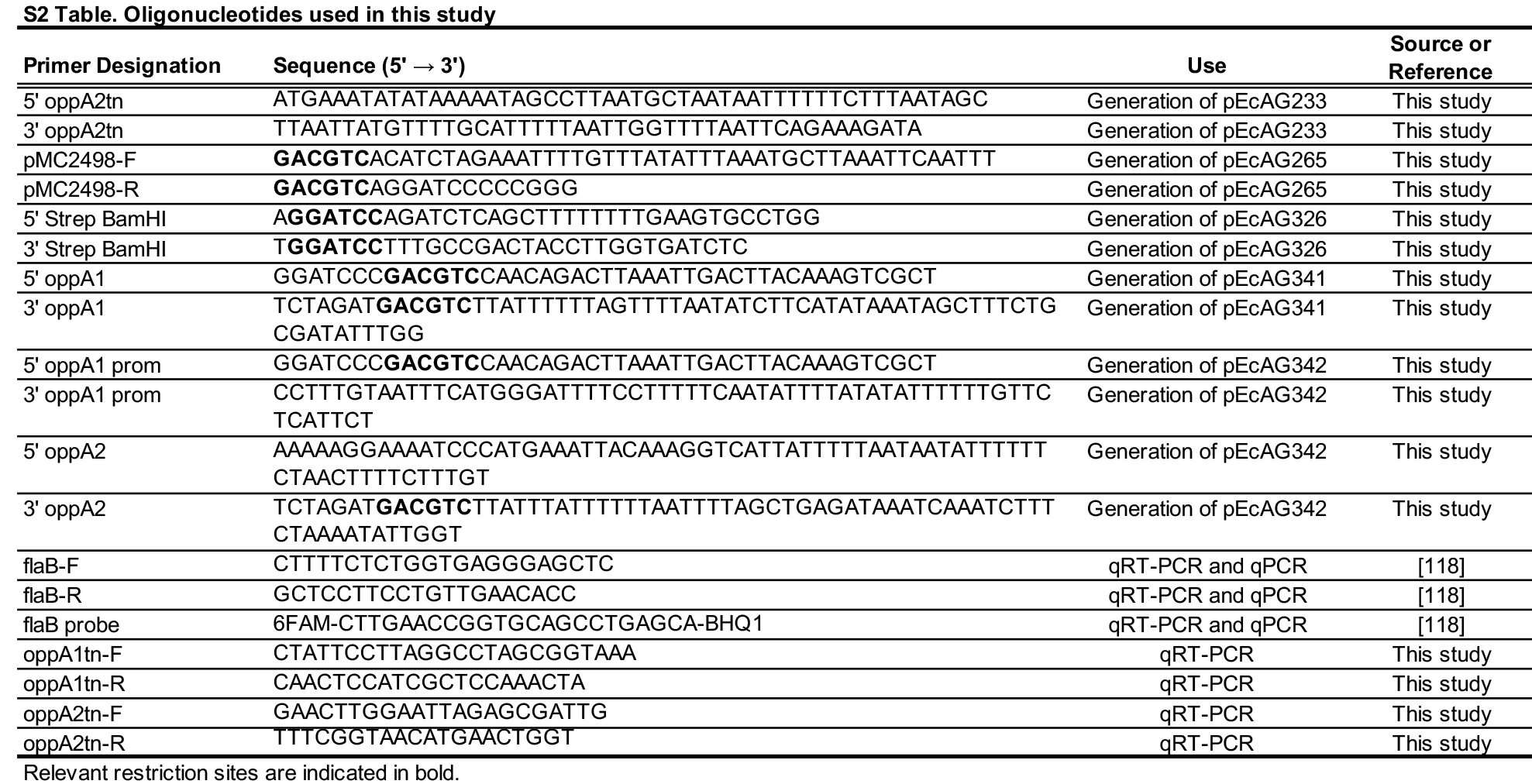

Supplement: S2 Table — (TIF) [file ppat.1009180.s008.tif]
